# Russian roulette with unlicensed fat-burner drug 2,4-dinitrophenol (DNP): evidence from a multidisciplinary study of the internet, bodybuilding supplements and DNP users

**DOI:** 10.1186/s13011-015-0034-1

**Published:** 2015-10-14

**Authors:** Andrea Petróczi, Jorge A. Vela Ocampo, Iltaf Shah, Carl Jenkinson, Rachael New, Ricky A. James, Glenn Taylor, Declan P. Naughton

**Affiliations:** Faculty of Science, Engineering and Computing, Kingston University London, Penrhyn Road, Kingston upon Thames, Surrey, KT1 2EE United Kingdom; University of Birmingham, Birmingham, UK; Hampshire County Council-Scientific Services, Hampshire, UK

**Keywords:** Supplement, Bodybuilding, Weight-loss, Health risk, Sport, DNP, Exercise, Illicit drug, Body fat, LC-MS/MS

## Abstract

**Background:**

2,4-Dinitrophenol (DNP) poses serious health-risks to humans. The aims of this three-stage multidisciplinary project were, for the first time, to assess the risks to the general public from fraudulent sale of or adulteration/contamination with DNP; and to investigate motives, reasons and risk-management among DNP-user bodybuilders and avid exercisers.

**Methods:**

Using multiple search-engines and guidance for Internet research, online retailers and bodybuilding forums/blogs were systematically explored for availability of DNP, advice offered on DNP use and user profiles. Ninety-eight pre-workout and weight-loss supplements were purchased and analysed for DNP using liquid-chromatography-mass-spectrometry. Psychosocial variables were captured in an international sample of 35 DNP users (26.06 ± 6.10 years, 94.3 % male) with an anonymous, semi-qualitative self-reported survey.

**Results:**

Although an industrial chemical, evidence from the Internet showed that DNP is sold ‘as is’, in capsules or tablets to suit human consumption, and is used ‘uncut’. Analytical results confirmed that DNP is not on the supplement market disguised under fictitious supplement names, but infrequently was present as contaminant in some supplements (14/98) at low concentration (<100mcg/kg). Users make conscious and ‘informed’ decisions about DNP; are well-prepared for the side-effects and show nonchalant attitude toward self-experimentation with DNP. Steps are often taken to ensure that DNP is genuine. Personal experience with performance- and appearance enhancing substances appears to be a gateway to DNP. Advice on DNP and experiences are shared online. The significant discrepancy between the normative perception and the actual visibility suggests that DNP use is-contrary to the Internet accounts-a highly concealed and lonesome activity in real life. Positive experiences with the expected weight-loss prevail over the negative experiences from side effects (all but two users considered using DNP again) and help with using DNP safely is considered preferable over scare-tactics.

**Conclusion:**

Legislation banning DNP sale for human consumption protects the general public but DNP is sold ‘as is’ and used ‘uncut’ by determined users who are not dissuaded from experimenting with DNP based on health threats. Further research with stakeholders’ active participation is imperative for targeted, proactive public health policies and harm-reduction measures for DNP, and other illicit supplements.

**Electronic supplementary material:**

The online version of this article (doi:10.1186/s13011-015-0034-1) contains supplementary material, which is available to authorized users.

## Introduction

Epidemics of obesity have been documented in most developed countries [1–3], thus finding efficient, but safe, pharmacological aids to weight loss is in the focus of the involved healthcare systems and the holy grail of obesity research. However, agents that are effective for weight-loss often have severe side effects [4]. Believing that freely-available supplements are not harmful, particularly when from a natural source, people often turn to herbal dietary supplements that promise weight loss by boosting metabolism and/or suppressing appetite. Nonetheless, commercially-available dietary supplements with weight loss claims have long been suspected for contamination with booster agents [5–7] and thus it was a conceivable assumption that DNP could be used as a booster agent in supplements sold as natural or herbal weigh loss aids.

From the public health point of view, it is concerning that hazards from the past (e.g., weight loss medications that have been stopped decades ago) seem to be a returning feature of today’s off-street supplement market, mainly through retail networks that fall outside standard safety regulations: the Internet [8]. One such drug category is the so called “rainbow diet pills”, representing an array of potent combinations of prescription medications, that are prohibited in medical practice but nonetheless available in disguise as herbal diet pills [9]. Another drug that recently returned to the underground weight-loss product market is 2,4-Dinitrophenol (DNP). DNP is a manufactured odourless yellow chemical that does not occur naturally in the environment. It was used in medical practice until the late 1930s to treat obesity and was subsequently withdrawn owing to its severe toxicity [10]. However, industrial uses of DNP as a dye, wood preserver, herbicide and film-developer have remained in place over the years. Recently, DNP resurfaced as a weight loss product in the supplement market as *Sulfo/Solfo Black, Nitro Kleenup* or *Caswell No.392* [11], but it also can be sold on the Internet or listed among the ingredients as *Aldifen, Chemox, Nitophen, Dinofan, Dinosan, Dnoc, Osmotox-, Fenoxyl-,* or *Tertosulphur PRB*. In parallel with the re-appearance of DNP as a weight-loss promoting agent, clinical presentations with DNP toxicity increased with associated high mortality worldwide [12–14].

To protect the public, the Food Standards Agency (FSA) issued a warning against health risks from potential DNP contamination of supplements to healthy individuals [15, 16]. Subsequently, the FSA took proactive steps to reduce the availability of DNP through Internet sales to UK customers [16, 17] and to ensure that DNP is not sold in disguise under fantasy supplement names. This project is part of the Agency’s effort to protect the general public from direct or inadvertent exposure to DNP at harmful concentrations; and the paper presents the results from the work conducted to this effect in collaboration between the FSA, local authorities, police and academic researchers between 2013 and 2015.

Counterbalancing the efforts of regulatory bodies, a culture exists among bodybuilders that encourages and provides advice for DNP use. Other than bodybuilders at risk groups are comprised of adolescents, extreme dieters and those who are suffering from an eating disorder, people with drug abuse history and athletes willing to experiment with dangerous chemicals [18–20]. It is important that health care professionals in frequent contact with these groups are cognisant about DNP-related risks and are well-prepared to recognise the possible signs and risk factors (i.e., desire to lose a significant amount of weight fast or to achieve extremely low body fat %) for early intervention. Equally, regulatory bodies and policy makers need to consider harm-reduction measures along with prevention through controlling access and warning against DNP based on risks to health.

### DNP as a weight-loss agent

**Fig. 1:**
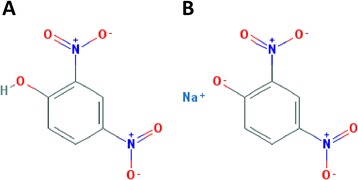
Chemical structure of 2,4 dinitrophenol and sodium dinitrophenolate (PubChem Open Chemistry Database)

Although not licensed for human consumption, DNP (Fig. 1a) and DNP crystal form (Fig. 1b) are used by bodybuilders and extreme dieters for their fat burning properties through inhibiting efficient energy (ATP) production in cells. Through uncoupling mitochondrial oxidative phosphorylation by facilitating proton transport across the mitochondrial membrane, DNP leads to rapid consumption of energy without generating ATP and consequently, to increased fat metabolism [11, 12, 21]. However, the weight-loss effect comes with serious, and in some cases potentially fatal, adverse side effects, namely hyperthermia (the leading cause of fatality with acute DNP toxicity) and cardiac arrest, but also diaphoresis, tachycardia, tachypnea, skin toxicity, Fourier’s gangrene and cataracts with low dose chronic exposure [10–14, 22, 23]. The proposed mechanism of DNP induced toxicity suggests the activation of ATP-sensitive K+ channels [24, 25]. The amounts ingested and length of exposure in DNP-related illnesses and deaths range between (1 to 46 mg/kg/day and 3 to 46 mg/kg/day, respectively). Taken together, the evidence suggests that there is no universal safe/danger zone with DNP. People who are not sensitive to DNP can tolerate a higher dose and/or longer exposure but the same dose is harmful to those with high sensitivity [26].

### Controlling DNP sales and consumption

Because DNP appears to be mostly obtained through mail order, controlling this problem from the regulatory point of view is a very difficult task. Under the Sale of Goods Act 1979, there are strict codes which sellers and retailers must abide by: the goods they sell must be as described, of satisfactory quality and fit for purpose. In 2013, the sale of DNP as a fat burner in the UK was made illegal due to health concerns [27–29]. As a result, those wishing to use the drug turned to the Internet and other sources. This makes regulation much more difficult as people have no or very limited legal or other recourse if the products are not effective or prove to be harmful [30], unless there are agreements to uphold the UK Law at the point of sale. As with online pharmacies [31], one key challenge in any country’s regulatory effort is the off-shore location, anonymity and fast-changing nature of Internet retail sites. Although DNP as a fat burner falls under similar regulation worldwide, it can be legally obtained for industrial use as long as it is clearly not intended for human consumption (i.e., not in capsules or tablets). This latter aspect makes controlling human consumption of DNP similar to the situation with legal highs [32, 33]. In both cases, the substance and advice on use are easily available through the Internet, where retailers and users are perceived as authoritative sources for information. Thus for effective prevention, it is imperative to focus on the users’ side with the view of developing an understanding of the underlying motivations and reasons for people’s willingness to take risks with uncut DNP.

With the persistent supply and popularity of DNP among bodybuilders and dieters, regulatory efforts to prevent DNP use are undermined by readily available retail options on the Internet. One alternative approach to mitigate health risks people willingly take with unknown, unlicensed and potentially dangerous substances is to devise end-user-centred, proactive public health policies. In order to do this, it is important to understand the factors that affect an individual’s willingness to take the drug after acknowledgement of the adverse side effects. Information on how and what people think about taking a risk with DNP for effective weight loss is mostly limited to the Internet accounts of users and those who are actively interested in DNP and seeking information though online forums and discussion boards. Apart from the Internet, one recent study in the scientific literature explored young adults’ willingness to take hypothetical risks with DNP for the desired result in weight reduction [34]. The results showed that vulnerability to DNP-risk was linked to having past experience with weight loss products and the magnitude of desired weight-loss. Albeit informative, and the first study to explore psychosocial factors relating to DNP-risk, the results were limited by the hypothetical nature of the study. Investigating psychosocial factors among those who are or have been taking DNP is vital to develop good understanding of the driving forces behind this specific health risk taking. The present study advances this knowledge by investigating-for the first time–the thought processes about DNP use among admitted users.

## Aims and objectives

The initial aims of the project were to assess whether regulatory efforts to prohibit DNP sale as a fat-burner agent have been effective and to ensure DNP is not available on the market in disguise, under some fictitious names as supplements to the public. Additionally, for the first time, the study looked at reasons and expectations associated with DNP from the users' point of view to inform effective prevention and harm reduction.

Thus the objectives of this multi-stage multidisciplinary study were:

(Study 1) to explore the online retailers and bodybuilding forums/blogs for availability of DNP to guide the sampling strategy in Study 2 by identifying bodybuilding supplements that may be DNP in disguise, may contain DNP as a booster ingredient (spiking) or prone to DNP contamination owing to poor quality management; and to inform Study 3 through personal accounts of experiences and informal advice given on how to use DNP and whether advice includes warning against DNP.

(Study 2) to investigate the level of risk from potential fraudulent sales or through adulteration/contamination in weight-loss promoter supplements; and

(Study 3) to examine DNP users’ motives and reasons for, attitudes about, normative perceptions and visibility of DNP use; along with purchase routes, experiences and risk-management practices with DNP.

## Methods

**Fig. 2:**
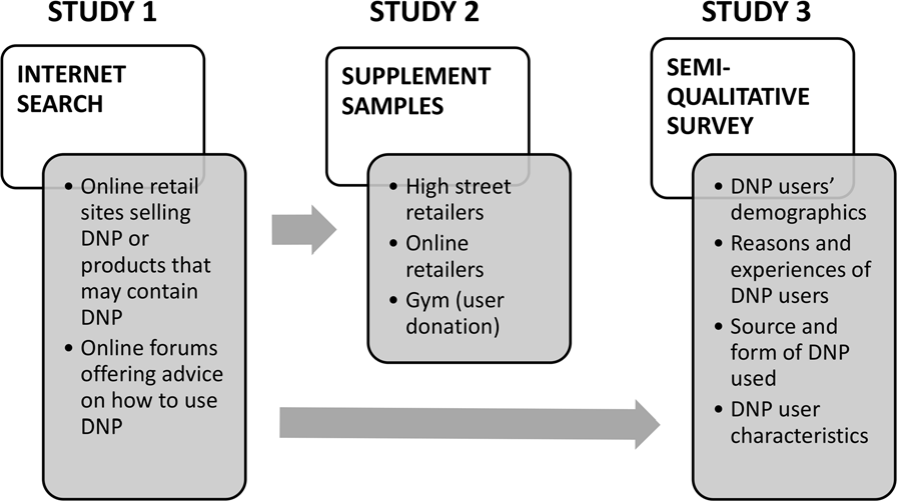
Flowchart of the research process depicting the three distinct phases of systematic Internet search (September - December, 2013), supplement sampling and screening (January - July, 2014) and survey study (December 2014 - August 2015). Internet sites were re-checked in March 2015 with the list revised in August 2015

The study consisted of three distinct phases. Study 1 investigated the Internet for the availability of DNP and advice on use. Study 2 focused on potential contamination or adulteration of bodybuilding supplements with DNP. Study 3 explored motivations, reasons, expectations and risk-management with DNP in a small sample of users. The flowchart of the research process is depicted in Fig. 2.

### Study 1: Investigating the presence of DNP on the internet

Informed by the research protocols for Internet research [35–37], the Internet search was conducted using the combination of “DNP AND bodybuilding”, “DNP AND sale OR retail OR buy” and “DNP AND fat burner OR weight loss” using two general global search engines Bing (https://www.bing.com/) and DuckDuckGo (https://www.DuckDuckGo.com), along with a meta-search engine “dogpile” (http://www.dogpile.com) which combines Google, Yahoo! and Yandex. Hits included into the database were limited to those available in English. Newspaper articles, YouTube videos and announcements posted on official websites of the regulatory bodies were excluded. Online retail sites were screened for whether DNP is offered ‘uncut’ or as an ingredient in supplements. Information recorded on these retail sites included formulation, recommended dosage, health warning (if any) and purchase options. For the forums and blogs, the hits were read in order and information on dose, cycle, diet, concomitant supplements/substances and reasons for use were noted.

Information available through both the retail sites and the bodybuilding forums on DNP was read in order of appearance of the sites on the search results until the harvested information was saturated (i.e., no new entry could be made). As the main aim of the Internet search phase of the study was to gather information to inform the subsequent studies (2 and 3), the focus of this phase was constrained to seven parameters, namely (1) availability, (2) formulation, (3) dosage, (4) cycle, (5) advice on use, including diet and co-supplementation and (6) health warnings (if any) and (7) reasons for use. When a supplement was identified as having potential for containing DNP, it was included in the supplement sample pool. To capture the dynamics of the supply side, websites identified initially were checked again at the end of the project, covering at least 12 months in between.

### Study 2: Screening bodybuilding supplements for DNP

Primary target products were dietary supplement, and specifically fat burners, that are marketed for performance enhancing and body-building, in all forms-including capsules, tablets, pills, powders and liquid forms. Protein shakes which may supplement fitness–particularly those with a long list of ingredients not just a protein were included. Meal replacements such as Slim Fast, Atkins, Slimsticks were excluded.

#### Sampling

Altogether, samples were procured from high-street outlets, gyms and from the Internet. Because Study 1 was part of an intelligence gathering project to assess compliance with current UK legislation for labelling [38], sample collection was conducted by Local Authority Enforcement Teams across England following an informal procedure for sampling and for the level of information recorded. Efforts were made to avoid duplication as much as possible, but duplicate samples were tested where received. Regarding supplement sources, Local Authority Enforcement Teams were instructed to exclude Internet retailers because these samples were obtained separately by the Hampshire Scientific Service. No specific exclusion/inclusion criteria were set for retailers because the focus of the study was on the potentially contaminated and/or adulterated products, not on retail outlets, but it was assumed that the likelihood of finding contaminated/adulterated bodybuilding supplement with DNP is higher in ‘less prominent’ retailers (i.e., independent stores, small franchise chains) owing to the limited resources available for quality assurance. Samples were securely placed in plastic containers to avoid leakage and catalogued using a unique alphanumerical identification code. There were no specific storage and transport requirements prior to analysis.

In addition, as part of the Health Weeks initiative (which is an annual event in February/March that raises wellbeing awareness and highlights the services that the university and the local community offer to support healthy lifestyles) students and patrons of a commercially-run gym located on a university campus were invited to submit an anonymous sample of the fat burner supplements they were using for free testing. Participants were instructed to place the sample into a dedicated, lockable metal box which was secured to the wall at the gym reception. Samples submitted via this method were accompanied by a brief questionnaire which asked participants to provide information on brand name and purchase location (at the same level of detail as for samples purchased from high street/Internet retailers). The information form also included questions on patterns of use, side effects experienced (if any), reasons for using fat burners (e.g., *as part of a fitness regime; for sustained weight management; as a quick start to lose weight/to lose weight for a specific occasion (e.g., competition, social event, etc.)* and *other*) source of information consulted about fat burners (“*What information source did you consult before you decided to use the product?*” with answer options of *product label; scientific publications; company website; retail shop information/leaflet; retail shop assistant; online retail product information; website forums and discussion boards; healthcare professional; athletic trainer; friend;* and *other*). The level of concern about the safety of DNP was rated on a 10-point scale with 1 = *not concerned at all* and 10 = *very concerned*. Participants were provided with the FSA 2013 statement on the danger of DNP [16] prior to their agreement to participate by donating a sample consisting of at least one daily dose in a sealed envelope and completing the short information form. One sample comprised of one unit of supplement (i.e., one tub of powdered supplement, one blister pack/pot of tablets, ideally a minimum of 5 g), complete with full labelling and usage instructions.

Where available, each sample was accompanied by photocopies of the original packaging and product labels. In addition, information available for these samples included the purchase location (county and whether it was high street vs. Internet retailer). These data, along with the semi-quantitative analytical results were entered into an Excel spreadsheet and processed using descriptive statistics and presented as bar charts.

#### Chemical analysis

For this phase of the project, the chemical analysis focused on screening the supplement samples for DNP using a liquid-chromatography-mass-spectrometry (LC-MS/MS) method which was developed in house. The LC–MS/MS system comprised of a 1260 infinity LC system (Agilent Technologies UK) with 1290 infinity thermo-stated autosampler, degasser, binary pump and column heater; coupled to a 6430 triple quadrupole mass spectrometer (Agilent Technologies UK). The ball mill used for sample preparation was Fritsch P23 mini ball mill, Germany. Further details of the method are provided in Additional file 1: DNP method development for semi-quantitative screening.

### Study 3: Investigating personal experiences with DNP among users

#### Theoretical framework

The main foci of the survey were on reasons for using DNP, along with personal experiences and satisfaction with the results and intention to use DNP again. Behavioural reasoning [39] offered a suitable framework through the exploration of reasons that explain planned, current and past behaviours. In behavioural models, it is always an interesting question how far (or near) objective data support the perception on which reasons are formed (e.g., perceived safety of DNP, particularly when one person’s ‘safe dose/regime’ is not transferable to another).

Notwithstanding, behavioural reasoning was used as a loose theoretical framework because it offers a practical insight into motivational mechanisms for taking risks with DNP. At this early stage of researching DNP-related behaviour and decisions, we did not set out to test a specific behavioural reasoning model. Rather, we aimed to explore–using a mix of quantitative and qualitative data–how users think about DNP to inform future research endeavours. The advantage of looking at DNP through reasoning is-similarly to other substances in the functional drug category [40]-that reasons incorporate pro/con explanations, cost/benefit rationalisation and situational facilitators/constraints and exert both indirect (through contributing to norms, attitudes and perceived control) and direct influence on behavioural intentions [39].

#### Sampling

Participants were recruited through Internet-based, English speaking discussion boards. The inclusion criteria were having personal experiences (current or recent) with DNP and willingness to disclose related information through the anonymous survey. Recruitment was done through posting a link to the survey along with a brief description of the study on discussion boards which allowed us to advertise this study among the members. We also asked participants and discussion board members to promote this study among their peers.

The final sample consisted of 33 males and 2 females with mean age of 26.06 ± 6.10 years (age range: 18–45 years). Owing to the worldwide reach of the Internet, participants were from the United Kingdom (*n* = 15), the United States (*n* = 13), Canada (*n* = 4), followed by Ireland and Panama (*n* = 1 each). The highest proportion in the sample was White/Caucasian (*n* = 25), followed by Hispanic/Latino/South American (*n* = 4), Black or African American and Asian/Pacific Islander (*n* = 2 each) and Arab (*n* = 2).

#### Measures

Informed choice in the health context is defined by meeting three conditions (being based on relevant knowledge, consistent with the values of the person and behaviourally implemented); and theorised that its extent is said to be assessed by knowledge about and attitudes toward the behavioural choice [41]. In the present study, a semi-qualitative, self-reported survey was administered using a closed online survey platform (surveymonkey) to capture DNP users’ value systems with five direct statements (response recorded on a 6-point Likert-type scale) about DNP use and users. The five statements were formulated based on the typical views propagated in the Internet forums. This direct approach offered a context specific focus on DNP among those who have personal experience with the substance.

To explore the context in which decisions about taking the risk with DNP were made, the survey also contained one question assessing the level of concern about the DNP product before use (recorded on a 10-point scale ranging from 1 = *not concerned at all* to 10 = *very concerned*); and whether DNP users have past and current experiences with fat-burners (“*Do you currently take or have taken fat burners?*”; recorded as yes/no) and past experience with illegal supplements (“*Do you have any past experience with illegal supplements?*”; recorded as presence vs. absence without specifying the type of substance). Normative estimation of DNP use was measured with a single question where participants were asked to estimate the proportion of bodybuilders who use DNP (0 % means nobody and 100 % means everybody). Along with the demographic details (age, gender, ethnicity and country of residence), information on height, current and ideal weight, current and ideal body fat percentage, weekly training times and type of gym used was recorded. Participants were also asked from what source and in what form they obtained DNP; and how they ensured (if they did) that the DNP is genuine and pure before they ingested and whether they used other supplements while using DNP.

#### Ethics, consent and permissions

The study was approved by the Kingston University Faculty of Science, Engineering and Computing Research Ethics Committee. Participation was completely anonymous and voluntary. Consent to participate was implied by voluntarily completing and submitting the survey. These conditions, including the implied consenting procedure and use of data, were clearly stated at the start of the survey.

### Data analysis

Data from surveys and analytical results were entered (Study 2) and downloaded (Study 3) into Excel spreadsheets. Data were cleaned and formatted for statistical analyses. Results from Study 1 were entered into a word document. Descriptive statistics (means and standard deviations) were used to show the data characteristics for the sample. Group comparisons were performed using independent samples t-test (*t*) and analysis of variances (*F*) or Wilcoxon test (*Z*) for nonparametric data. Relationship between the reported and estimated DNP user numbers was tested using Spearman *r*. Associations between categorical variables were assessed with chi-square test using Fisher’s exact tests for significance. Statistical significance was set at p < 0.05. Following the reporting guidelines for statistical tests in medical and behavioural research [42, 43], respective effect sizes (*d*, *partial eta squared* and *w*) were reported for all non-significant statistical tests and means and standard deviations were provided for all comparisons.

The magnitude of the effect was discussed according to the reference values recommended by Cohen [42–44]. Effect sizes were discussed in both theoretical and practical implications. Assuming that the small sample size, coupled with large standard deviation, result in a loss of power in cases where the effect size is small to medium, we also reported detailed information to enable a priori calculation of the required sample size to reach statistical significance at the conventional 0.05 and the newly recommended 0.005 levels [45] to inform future research and facilitate replicability.

Statistical analyses were performed using IBM SPSS v22. Respective measures of the effects were obtained from the SPSS output where available or calculated using the online meta-analysis effect size calculator [46].

Qualitative responses were analysed using applied thematic analysis [47], following the guidelines set for thematic analysis in psychology [48]. Applied thematic analysis was selected as analytical framework for the qualitative responses because it is an inductive procedure that supports theoretical model development and finding solutions to real life problems. It is a positivist/interpretative approach in a sense that assertions must be supported with evidence (data). Applied thematic analysis is particularly suited for the data at hand because it allows quantification and inclusion of text of any length (i.e., survey questions can be treated as text) and facilitates studying a particular topic (users’ views of DNP safety, effectiveness and risks) rather than individual experiences.

## Results and Discussion

### Study 1: Information available on DNP from the internet

The internet search, after removing duplicates, yielded 92 websites of which 36 supplied DNP at the time of writing this article (August 2015). The search also identified a significant number of retailer websites which are no longer available. Details of the websites are provided in Additional file 2: List of retail web-based sites and discussion boards/forums. A comprehensive list was not attempted because of the fast changing nature of the DNP market. Rather, it is a snapshot of the situation illustrating the abundant supply and informal unchecked information available on the Internet. A considerable number of online retail sites (trading in anabolic steroids and other bodybuilding supplements) that do not sell DNP still provide information on DNP including a reference to alternative availability (e.g., from the UnderGround Lab). Forums are characterised by multiple threads started on various aspects of DNP use. These discussion threads typically initiated with an enquiry about DNP availability and use. Basic information; dosage; supplementation; side effects and allergic reactions; concurrent use of other bodybuilding substances and experiences are readily shared but responses tend to be void of or explicitly refuse suggesting retailers. Information on DNP manufacturers is shared among users, again, often initiated by someone with intention to purchase a particular brand. Independent of the legality of the transaction, word-of-mouth quality assurance and customer endorsement appears to be a prominent features in building trust for online purchases of DNP, which is in line with the general literature on online buying [49–52].

Detailed diary-like blogs on daily experiences with DNP are fairly common. Most of these blogs offer detailed accounts of the DNP ‘self-treatment’ regime, along with users’ expectations, fears and hopes, and experiences. Similarly to the phenomenon observed in the community of *psyhconauts*, the educated and informed recreational drug users [53, 54], lived experiences with DNP are often translated into advice for others who wish to experiment with the same substance. Without exception, these advices about DNP use aim to make the experience–risks acknowledged–better and safer.

#### Online availability of DNP

The incongruence between the geolocations of the *ip* addresses and shipment information (where available) indicates that DNP suppliers 1) possibly hide behind layers of web-pages; making policing difficult and 2) use multiple websites simultaneously. In some cases, DNP is sold with health warnings or a statement that the sale is not for human consumption and “*packed in capsules only for means of safe transportation*” (quote from the Q&A section of an online retail site), but the overall impression of the retail sites is that DNP is openly sold as an effective fat-burner/bodybuilding supplement.

Images on retail sites either show the compound as powder, capsules or pills; or the packaged product with manufacturer’s labels. Manufacturers, when identified, include Biomax Lab (Turkey), BR Europe which is now UmForte (US), BodyAdvance Performance (US), Gen-Shi Pharmaceuticals (Japan), AbaXen Pharmaceuticals (US), Aeolis Pharmaceuticals (US), TrigoPharm (The Netherlands) and Wildcat/British Research Laboratories (UK). DNP is sold and labelled ‘as is’ with only one exception found. The pack from Gen-Shi Laboratories shows L-Glutamin but the retail site clearly identifies the product as DNP, giving the compound name, manufacturer, dose, quantity. In addition to online retail stores, D-Hacks Lab (UK) and Crystal Heat Labs (UK) supply DNP through Facebook. Concerning mismatch in information on wholesale sites is not uncommon. For example, one retail site depicts DNP as yellow powder but with “steroid powders or liquid” overlaid on the image and discusses the recreational use of an entirely different substance, dextromethorphan (a cough suppressant), beneath the image under Application.

On most online retail sites, purchase requires registration and setting up a customer account. Many of them accept major credit cards. Alternative payments include bank transfer, Western Union, Moneygram or ‘bitcoin’. In terms of getting the DNP orders to customers, online retailers pride themselves for high success rates going through customs and offer a replacement guarantee (free replacement once with proof for the seized package) for most countries but Australia, Canada, New Zealand, Singapore and South Korea where only one shipment is made. Under shipping information, retailers explain in detail that they avoid identifying themselves as pharmaceutical-related or labs to avoid suspicion, using discreet packaging that does not identify the content and employ frequent changes to their packaging not to alert customs (i.e., using non-descriptive labels or disguise DNP as some other product, e.g., turmeric if DNP is shipped in powdered form). When specified, shipment is often made through Turkey. One typical example is:

“*We have a success rate of 98 % at USA, Canada and European customs with Registered Mail Method only. Our company eliminates the remaining 2 % risk and gives you a customs seize guarantee. If your order is seized at the customs, we ship one more time for free. Seizured orders must be sent to a different address because customs may flag your address. And the best part is you do not have to pay the shipping charge again. We don’t have any other options of refund. We cannot resend seized orders to Australia because of extreme custom security. Orders from Australia are welcome but no one can give a %100 guarantee no matter how professional and safe packing is done*”.

For obvious reasons, online retailers do not openly disclose how it is achieved but as one user said: “*trust me, anyone could open the package and they would not have a clue*”.

#### Online accounts of experimenting with ‘uncut’ DNP

The informally “recommended” dosage ranges between 200 and 600 mg, often recommending that new users start at a lower dosage and increase it gradually if tolerated well. For comparison, 100 mg equates to 1.4 mg/kg for healthy average male (70 kg); 1.7 mg/kg for healthy average female (58 kg); 1.2 mg/kg for average bodybuilder male (81.5 kg); 1.6 mg/kg for average bodybuilder female (63.5 kg), based on ideal weight for the average height, with ideal weight adjusted for bodybuilding [55, 56]. Health warnings are associated with a single dose (i.e., it should not exceed 300–600 mg at any one time) and duration of the DNP regime. The latter varies between recommended cycles (i.e., 8 days on-8 days off; 7–10 day cycle; 2 weeks on–2 weeks off; 20 or 30-day cycle, with shorter cycles tending to be recommended with higher doses) and total duration (i.e., do not exceed 20 days). According to some accounts, other substances with similar effects such as Clenbuterol (a bronchodilator with fat burning properties) are sometimes also taken in combination with, or in between, ‘on’ periods. Taking antihistamines (e.g., Benadryl or other allergy medications, quercetin extract) to manage allergic reaction is suggested, along with vitamins and stimulants such as caffeine. Kratom, a plant with stimulating effect at low to moderate dose [57], which makes monotonous hard physical work more bearable, is also gaining popularity among DNP user bodybuilders; and it has been speculated that it could counterbalance the lethargy caused by DNP. Other substances discussed for use alongside DNP include appetite suppressants, thyroxine (T3) and insulin-with mixed views and explanations.

Most concerning is that in the absence of easily accessible information and a universal safe zone for dose and exposure duration, this individual, personal-experience based advice for length and pattern for the ‘treatment’ regime is highly concerning for the public. None of these information sources draw people’s attention to the fact that DNP’s harmful effects are highly dependent on the individual’s tolerance. The only recommendation is to start at a lower (200 mg per day) dose and increase if tolerated well. Based on the available scientific evidence, even this lower dose can be harmful for some [26].

Critically important from the public health point of view are the patterns of use by bodybuilders and extreme dieters-as recorded on Internet blogs and forums - which provide an overwhelming body of evidence that dedicated users take the ‘uncut’ compound (in tablet or crystal form). Thus, the issue is not only that DNP might be sold in disguise; nor is the contamination with DNP and the trace amounts that regulatory bodies need to be worried about, but the easy access to the pure compound. The potential consequence of this is that pure DNP may not only be purchased and consumed by the dedicated and highly experienced bodybuilders who are well-prepared, but it is just as readily available to the naive users who lack experience in taking risky substances.

Several postings were concerned with the legality of obtaining DNP. These discussion threads tend to be initiated by someone interested in using DNP as part of their information gathering. Responses to such inquiries depend on the country where the responding expert user resides. Users are cautious about new brands and seek reassurance for the quality and effectives from other users.

Most notably, a plethora of postings exhibit remarkable in-depth knowledge of biochemistry which manifests in postings explaining the mechanism of DNP to novice users as well as in postings discussing synergistic effects between DNP and other substances. Reference to scientific literature is not uncommon but these postings tend to come from the same members who are most likely with relevant educational background. Experienced forum participants make efforts to provide detailed and informative answers, and take pride in their responsible and considerate approach. Disapproval of DNP use is not uncommon suggesting that although DNP use is prevalent among bodybuilders and avid exercisers, it is not a universally accepted and approved practice. In fact, those contemplating DNP use and seeking information on how to use DNP are often vetted for dietary habits, exercise regime and previous experience in controlled weight-loss and maintenance before advice on DNP is offered. In this tightly-knit, self-regulating community, novices who take this substantial, knowledge- and experience-based but impartial advice lightly or discard the warnings are often scolded for their cavalier attitude and brassiness. Questions that show absolute lack of investment into acquiring knowledge (e.g., *“is DNP safe?”*) or effort in weight management through diet and exercise are not well received and rebutted with a quick judgement that the person is *“*not ready*”* for DNP.

Discussion forum members sharing their experiences with DNP appear to be well-informed about the different forms such as tablets, crystal (sodium dinitrophenolate); or industrial grade powder; and their strength. These users are confident and appear to be highly experienced in using a cocktail of performance enhancing substances and are accustomed to dealing with (temporary but often harsh) adverse effects. Although accounts of death in this experienced community appear in the Internet postings, the fact that the majority of users survived and are often satisfied with the outcome of their DNP regime gives the impression to the public that DNP may not be as dangerous as the scientific literature and official standpoints indicate. However, the fact that these experienced users have managed to use DNP “safely” (that is, using DNP without death or apparent indication for long-term health consequences) does not necessarily translate to “safe use” for others, particularly among drug-naive users or those with minimum experience. On the contrary, it creates an illusory sense of safety which - coupled with a potential lack of knowledge about the strengths of different forms and the complete lack of regulatory control over these substances and labels - indeed poses grave danger to the general public. Furthermore, owing to the lack of established level of toxicity, a dose well-tolerated by one user is not applicable to another.

Owing to DNP use being a rather clandestine activity (i.e., difficult to find DNP users in ‘real life’ settings), researchers use discussion boards to recruit study participants, the present study included. Another recent example appeared briefly in March 2015 on one discussion forum, comprised of a brief “survey” aiming to explore body temperature related personal experiences in support for research investigating the potential therapeutic effect of DNP in treating hypothermia. The survey lasted less than 5 hours before the discussion thread was locked by the moderator and thus only generated a limited number of, but intriguing, responses. Forum members’ responses exhibited a great degree of suspicion, as well as support for research and remarkable knowledge about DNP. The extreme difficulty of finding people who have tried DNP - particularly in real life settings - and willing to admit DNP use was openly acknowledged.

### Study 2: Screening results for DNP in bodybuilding supplements

A total of 98 samples were collected as follows: 77 were purchased in high street stores across the South of England, 16 were purchased online and five samples were donated through a local gym (of which two originally obtained from Internet, two from high street retailers and one from a gym). All five participants who donated a sample through the gym identified sustained weight management as the goal, with both being part of a fitness regime and as a quick start to the desired weight loss also appearing in two of the seven cases. Information sources were wide-ranging but notably only one person mentioned healthcare professionals (among other sources). The analysis found two of the six samples positive for DNP. Both were reported as purchased from UK Internet sites. Although side effects were reported (shaky feeling, anxiety and heart racing), these were not for the samples that were found with DNP. All participants expressed a high level of concern about DNP.

**Fig. 3:**
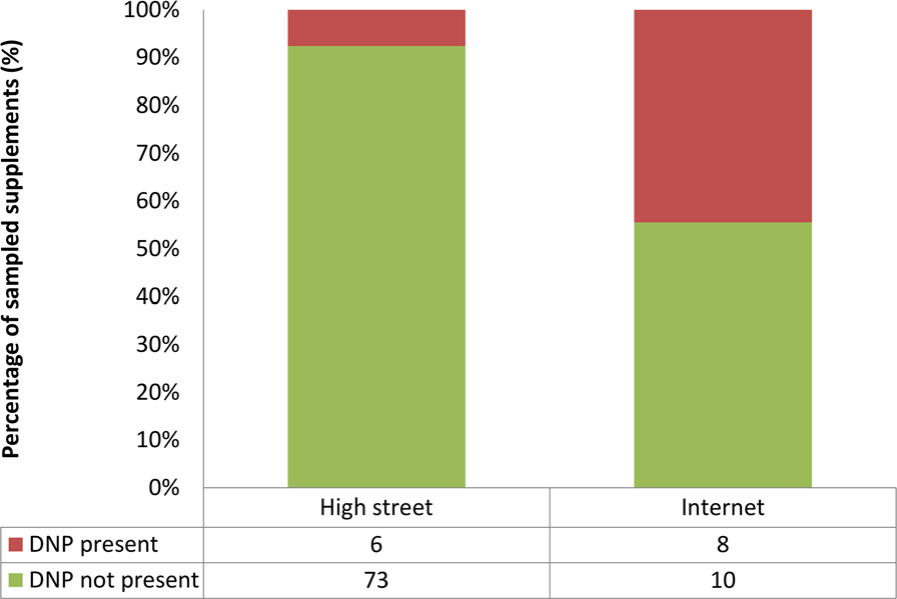
Proportion of samples contaminated with DNP obtained from high street and online retailers

Of all samples, 14 were found positive for trace amounts (<100 mcg/kg) of DNP (Fig. 3). In contrast to high street retailers, a concerning proportion of the Internet samples were contaminated with DNP. Because DNP was detected using semi-quantitative methods (for details, see Additional file 1: DNP method development for semi-quantitative screening), the results did not afford comparisons of the concentration between different retail options; nor was it practically meaningful owing to all DNP levels being detected as trace amounts. Further interpretation of the results would warrant a study using a larger sample size and a fully validated quantitative method.

Positive cases were reported to the Food Standards Agency through official channels. The low concentration of DNP suggests contamination owing to poor quality control rather than deliberate adulteration (‘spiking’) to increase effectiveness. However, the fact that DNP contamination in these supplements could occur in the first place suggests that the actors of this market chain (i.e., manufacturers or distributors) of the contaminated supplement were also in contact with DNP.

### Study 3: Personal experiences with DNP among users

The majority of the participants reported that they exercise three times (*n* = 16) or more (*n* = 15) a week. The remaining minority (*n* = 4) do not exercise or exercise only once per week. Seventeen participants use commercial gyms that are part of a chain, nine visits bodybuilding focused gyms and seven identified the gym they use as sport focused (2 responses were missing). DNP users’ profiles are summarised in Table 1. Seven of the participants (one female and six males) reported a desire to gain weight; two males with current use of fat burners. As expected, no participants in this sample wanted to increase body fat percentage. It is notable that despite the considerable variations in the current body fat percentage, males in all three age groups aim for an average body fat around 9-10 % (range = 6–15 %). Means and standard deviations for each group are displayed in Table 1. Themes emerging from the accounts of DNP users’ experiences are quantified in Table 2. Different sample sizes are due to missing responses.

**Table 1:** Participants body mass index (BMI) and body fat profiles by age groups^a^

		Male			Female
		18–25 years	28–36 years	39–45 years	18–25 years
	N	22	9	2	2
Body Mass Index (BMI)	Mean	27.75 ± 3.70	30.82 ± 3.82	30.85^b^	21.14 ± 3.11
	Range	21.5–35.6	26.2–35.2	-	18.9–23.3
Actual body fat (%)	Mean	15.89 ± 4.05	21.83 ± 14.23	14.50 ± 2.12	26.50 ± 2.12
	Range	7–25	12–50	13 and 16	25 and 28
Desired body fat (%)	Mean	9.84 ± 2.06	9.69 ± 2.06	9.00 ± 1.41	19.00 ± 1.41
	Range	7–15	6–12	8 and10	18 and 20

^a^Age groups were created using k-means clustering maximising distance (F(2,30) = 122.506, *p* <0.001; all pairwise differences are *p* <0.001). Differences in BMI and body fat percentages are not statistically significant

^b^missing value

**Table 2:** Summary of users’ qualitative responses for reasons, expectations, experiences and steps taken to ensure quality of the product, presented in decreasing order of frequency

Categories^a^	Content	Frequency
Reasons	Effective/fast fat loss as the ultimate goal	20
	Effective/fast fat loss to enable working toward the ultimate goal	4
	Curiosity	4
	Image enhancement	2
	Increase calorie intake without consequences on weight	1
	Shortcut	1
	Recommendation	1
Expectations	Positive outcome	13
	Specific side effects	6
	Mixed positive outcome and generic unpleasant experience	5
	Mixed positive outcome and specific unpleasant experience, including death	3
	General unpleasant experience	2
	Nonspecific responses	2
Ensuring quality	Trust and reputation of the dealer/retailer	16
	Self-experimentation	7
	Analytical testing	6
	None	4
	Visual examination	2
Experiences	Weight loss and side effects were as expected	16
	Reality adjustment	4
	Negative experience with side-effects	3

*Notes:*

^a^categories emerged from the data

#### Reasons

Reasons by which participants retrospectively justified using DNP were dominantly related to a desire for an effective weight-loss strategy - either as an end-goal or as means to an end-goal, as exemplified by reasons identified as: *“Just looking to drop some fat quickly”*, *“to be able to accomplish my goal in 2–3 weeks”* and *“the opportunity cost of time and the realization the DNP can shrink my cutting time by 2/3”*. Curiosity (*“to verify the hype”*) and image enhancement (*“recomp[osition]”* and *“improve image, and tone up”*) were also mentioned more than once.

A smaller, but not insignificant group, rationalised DNP use as a feasible option after trying for other ways to manage weights. One participant said:

*“I have lost over 60lbs in the past 24 months, yet still have some very stubborn fatty areas. No matter the level of cardio or calorie intake I cannot shift this fat. … DNP can literally and effectively burn fat from your body in a limited time period. As you can imagine this appealed to me.”* (23 year old male)

Another participant reasoned:

*“At 45 when I eat less my metabolism slows to compensate. I have to starve myself now to lose fat. Fasting and low carb dieting together resulted in a lot of muscle loss with any fat loss. My body seems programmed to preserve fat! DNP allows me to lose weight on a mild diet that my body would otherwise just adapt to by reducing metabolism and increasing catabolism. I am able to keep my metabolism at 100 % or above while eating less resulting in muscle sparing fat loss. This has been of great benefit to my overall long term health, despite the mild side effects during my DNP cycle.”* (45 year old male)

Both of these users experienced that diet and exercise alone did not yield the desired fat loss and thus perceived a need for chemical help. Despite that DNP is often classed with other diet pills as shortcut that replaces hard work, exercise and diet [58, 59], only one participant justified DNP use as a shortcut: *“to lose weight the easy way, can be very lazy sometimes”*.

Interestingly, one of the two female DNP users rationalised DNP use as a countermeasure for the calorie intake. She said that using DNP would allow increased calorie intake without consequences on weight: *“wanted to slightly increase the amount of calories I consumed while still losing weight”*. Such use of DNP is akin to the use of diet pills by those with eating disorders as a compensatory behaviour [60–62]; and also noted as a contributor to Eloise Parry’s DNP-overdose [63].

#### DNP use patterns and experiences

The quantitative results showed that the majority of DNP users (*n* = 25) have had previous experience with illegal supplements other than DNP. Thirty-one DNP users reported concomitant supplement use. The type of gym they visit and past experience with illegal supplements (*χ*^*2*^(2) = 1.188, *p* = 0.595, *w* = 0.182) or concomitant supplement use were unrelated (*χ*^*2*^(2) = 1.196, *p* = 0.405, *w* = 0.307). Half of the sample (*n* = 18) reported current use of fat burners with type of gym being unrelated (*χ*^*2*^(2) = 2.868; *p* = 2.77, *w* = 0.311).

For most respondents, DNP fulfilled the expectations without notable side effects. Such positive experience is exemplified by the following quotes: *“lost approx 1 lb of fat per day”, “I cut 35lbs of fat within a month”* and *“rate of fat loss and sweating were exactly as reported, even better didn’t die luckily”*. Others had mixed feelings about DNP. One 23-year-old male exerciser who used DNP for image improvement said the DNP *“did the job but [with] horrible side effect like headaches”.* Another user noted but immediately mitigated an unwelcome effect on diet: *“cravings were unbearable so diet suffered but didn’t gain any fat”* (28 year old male)*.* Many others reported a degree of reality adjustment between expectations and experiences: *“Had plenty of weight loss- wasn’t as easy as I had hoped”* and *“the weight loss is easier, but not as easy as some claim”,* or even *“less weight loss than expected”*. The self-reported symptoms were irregular heartbeat, nausea, vomiting, agitation and insomnia, which are in line with those reported in the literature [12, 13].

A few participants said that the side effects were less taxing than what they were prepared for: *“side effects seem largely overstated”* and *“I did not get as hot or sweaty as I expected, but this may be because it is winter and relatively cool everywhere”*. One participant explained:

*“… for me 125 mg/day was enough to lose 1 lb of fat/day. Most of the day I didn’t notice the side effects. At night or in a hot room it was uncomfortable due to the sweating and heat but normally I would not notice the side effects”* (33 year old male)

Accounts downplaying the side effects - if made on open Internet forums - may inadvertently underplay the harshness and potential danger of the drug. In reality, toxic effects vary widely between individuals and thus positive cases cannot provide reassurance, or guarantee, for others having an easy DNP course. Among users it was notable that despite the negative experiences with side-effects (e.g., *“[DNP] caused allergic reaction”* and *“DNP made it hard to catch my breath or more specifically it was harder to expel CO*_*2*_*”*), all but two male DNP users would consider using the substance again. Both participants who said that they would not use DNP again were identified curiosity (“*see if it works*”) as the main reason for using DNP in a first place and perhaps saw DNP as one alternative to the desired weight-loss. Even if both said they wanted to lose weight and they were satisfied or to some degree satisfied with the results, they expressed no intention to use DNP again. In the small subsample of those who highlighted negative side effects without being specifically prompted (*n* = 5), there was no clearly observable pattern between the forms or sources of DNP but apparently affected their satisfaction with the drug (all reported that they were somewhat satisfied with the results). Notably, 48.6 % of the sample (*n* = 17) felt that their expectations about DNP were fulfilled with the remaining 51.4 % (*n* = 18) reporting fulfilment to some degree. This relatively positive view, along with qualitative evidence suggest that users were knowledgeable about the drug and its desirable and undesirable effects and were prepared for dealing with the unpleasant side effects. As one participant summarised:

“*I had fully researched DNP side effects, including death. I expected extreme heat and sweating, lethargy, headaches, staining of bodily fluids and skin (yellow). I expected to lose 1 lb per day.*” (23 year old male)

Negative experiences were equally related to effectiveness as a weight loss agent and harsh side effects.

#### Access to DNP

The anonymous Internet purchase was the dominant method of obtaining this unlicensed drug (n = 26), of which only one was through the “dark web”, SR2. In only 6 cases users reported that the DNP was obtained from a fellow bodybuilder and only one each identified ‘dealers’ and ‘shop’ as the source for obtaining DNP. Almost all of those who identified the Internet as a source for DNP checked out the reviews about the retailer before (e.g., *“used a very trusted internet source”*) or made purchase based on recommendation (e.g., *“[DNP] was from well recommended source”*).

Reputation appeared to be an important feature for ensuring trust in the quality and relative safety of the substance, expresses as the *“great reputation of the guy” or “reputation: reviews from others on internet forums”*. DNP users’ responses suggest that they tend to rely on customer reviews (e.g., *“I ensured that I got it from a reputable source with lots of good reviews”*) and word-of-mouth (e.g., *“[DNP] was from well recommended source”*) when purchasing from a website. When buying from a seller, as opposed to an impersonal online retail site, personal past experience (e.g., *“I trusted the supplier” and “trusted source on other matters”*) was the most often cited way of ensuring that the product was genuine. Among the responses DNP users offered, there was some indication that suppliers of DNP are also users, which has helped to build reputation and ensure trust in them and in their products (e.g., *“I had the same symptoms as a previous dealer I went through”*).

Despite the regulation that DNP cannot be sold for human consumption, in most cases, DNP was in capsules (*n* = 26), with some purchased in tablet form (*n* = 3). This is consistent with the results from the Internet search which showed the DNP sold in online shops is typically in 100 mg or 200 mg capsules. Only in 6 cases DNP was purchased in powder form, which is how DNP is typically used for industrial applications. No association was found between the form and source (*χ*^*2*^(8) = 13.176, *p* = 0.094, *w* = 0.615).

#### Concerns and risk management

All 35 users in our sample knew the health risks associated with DNP intake and made an informed choice. Characteristically for the sample, DNP users were not only aware of, but were also prepared for or even took pro-active steps to manage the expected side effects. Participants typically said that *“I was on top of water and electrolytes” and “[I expected to] feel ill, heat, dehydration. I planned ahead for all foreseeable side effects.”*

The level of concern about the quality of the DNP varied widely among the DNP users. The concern about DNP in this context refers to whether the substance purchased ‘underground’ was of high quality, unadulterated and pure. Some participants tested or get the substance tested before use. One respondent, who later stated that he did not use DNP after all, said: *“I ran it through an HPLC and compared to Sigma-Aldrich 99.9 % DNP”*, whereas others sent the DNP off for laboratory testing: *“I used WEDINOS substance testing service (Welsh health board run service) which confirmed DNP”* and *“third party testing”*. (Note: to date, WEDINOS [http://www.wedinos.org] has recorded 4 samples with DNP as major ingredient in powder, pill, crystal and capsule format with one each, all submitted in 2014).

Half of the sample, however, relied on reputation of the seller/retail (*“ensured that I got it from a reputable source with lots of good reviews”)*. Others physically examined the DNP capsules or pills: *“Looked for the characteristic side effects and opened a cap, saw yellow crystals”*. Using one’s own body to test was not uncommon. A quarter of the participants reported some form of self-experimentation for testing DNP (e.g., *“after taking a few doses the side effects matched up exactly, and there isn’t much else that gives those specific sides”* and *“[I relied on] reputation, plus I had the same symptoms as a previous dealer I went through.”).* As one DNP user explained: *“I opened that capsules and looked at the crystals. I took the pills and started feeling the side effects. There was nothing else I could do to ensure the quality, but it was bought from the most reputable seller at the time.”* Only four users said that they took no action at all to ensure that DNP was genuine.

#### Attitudes

**Fig. 4:**
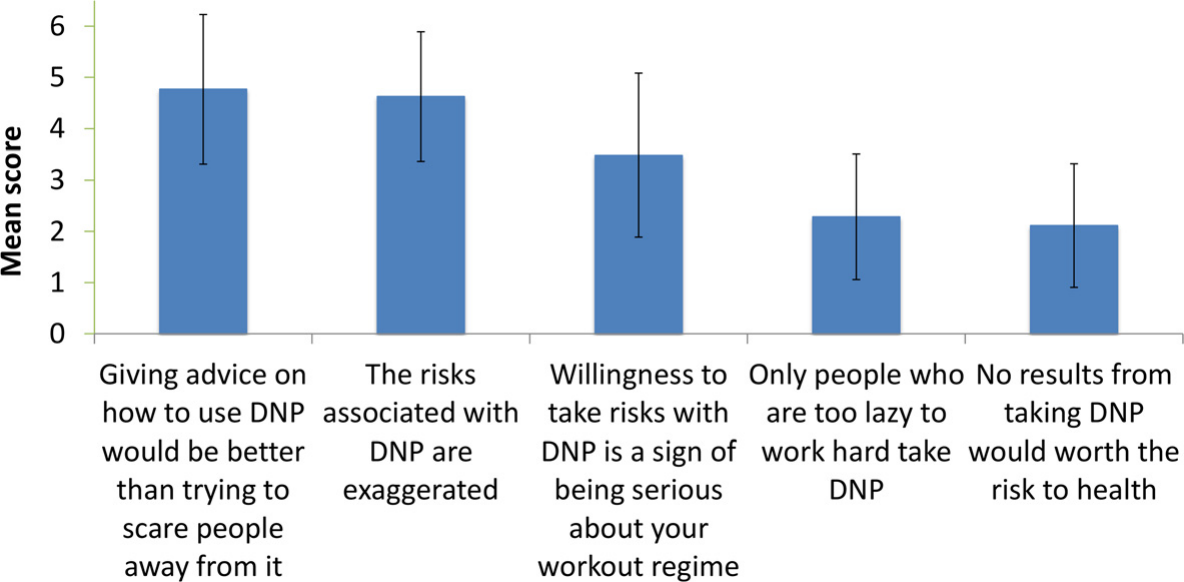
Users’ attitudes toward DNP and prototype perceptions of DNP users

Attitudes toward DNP use and users were quantitatively measured with five statements (Fig. 4). Cumulatively, the attitudes which users hold about DNP suggest that DNP use is considered a sign of being committed and risks with DNP are seen as ineffective “scare tactics”. The strongest agreement (4.77 ± 1.46 on a 6-point scale where scores 4, 5 and 6 indicate a degree of agreement) was recorded for getting help with advice and medical supervision instead of scare tactics.

Qualitative responses provided overwhelming support for the explicitly expressed demand for accurate and impartial information on DNP. As one participant (31 year old male) said: *“there really isn’t much out there for DNP aside from 1930s studies, anecdotal stories, and ‘bro science’”,* identifying the Internet forums as main information source.

*“There’s a lot of information on DNP on forums, however if there were more information about how to use DNP would be much better on respectable sites. All academic paper speak badly about DNP and how it’s a pesticide but it has more uses as a fat burner.”* (22 year old male)

Notably, in those who ask for more information, the danger of DNP is clearly acknowledged and not the drug but users are blamed for detrimental health consequences:

*“DNP is not right for everybody, but it was and is the best diet aid I’ve ever used. Proper understanding of the effects of DNP is necessary for the proper application of it. Any tool can be a weapon of self-destruction in the hands of a fool. Don’t outlaw tools just because fools will misuse them. DNP is already hard enough to get for those who are benefitted by it. I feel DNP is a far superior solution to surgical intervention for those who have difficulty losing weight or have devastating metabolic syndromes that modern medicine fails to allow DNP to be used to treat. A quick drop of 50 pounds would do your average diabetic person a world of good! DNP is a relatively effective non-invasive tool to do that. Don’t throw the baby out with the bath water! Far more people have died from obesity and metabolic related syndromes or had decreased quality and length of life than will ever be lost to DNP abuse. DNP has the power to do so much good in the hands of caring health practitioners and educated citizens.”* (45 year old male)

Another participant explained:

*“DNP with the right education and supplementation is fine! However, people are stupid. If you can die from something, people WILL die from something. DNP is no exception. However to the educated people who research, DNP is an effective supplement.”* (31 year old male)

Media reports on DNP-related death cases appear to support this argument showing that many DNP deaths were caused by reckless use or incidental overdose; either by young individuals who suffer from some form of eating disorder or inexperienced users [63–66]. One of the most recent examples is the tragic death of Eloise Parry who apparently took eight diet pills containing DNP [63]. Following this incident, in connection with its Operation Pangea [http://www.interpol.int/Crime-areas/Pharmaceutical-crime/Operations/Operation-Pangea] - investigation targeting online sales of pharmaceutical substances - and in collaboration with the World Anti-Doping Agency, the Interpol issued an Orange Notice warning about DNP as a potentially lethal diet aid [http://www.interpol.int/News-and-media/News/2015/N2015-050]. However, users’ views on DNP show a stark difference to the official stand. As one 22 year old male said:

*“DNP, in itself is a fantastic drug… There is nothing that can even come close to the results. Now granted it is a dangerous drug. It’s extremely easy to OD [overdose] on the shit and there’s no reversal. That typically scares people, which is why the FDA banned it. Human analogy suggests that if something works, take more. That isn’t the case with DNP.”*

DNP was considered as a very potent and useful drug by many users in this sample: “*DNP is highly effective and low doses work the best while avoiding highly-oxidative and side-effect inducing problems*.” (23 year old male). The danger in overdosing and from the side effects was readily acknowledged but also counter-argued that *“Any drug is dangerous if misused, info on the right dosage would be beneficial”*.

Reference to alcohol and tobacco - the two substances typically used as baselines for any supportive argument for relaxed drug regulation - also appeared in the supportive argument for DNP:

*“I believe this chemical is dangerous but with proper precaution the side effect and danger is worth the risk. I do not consider it any more dangerous than alcohol or cigarettes. I believe medical authorities should research this as a partial cure for obesity. Under the supervision of a medical professional I believe this could improve the quality of life of millions.”* (33 year old male)

Some participants exhibited a nonchalant attitude toward one’s own body, health and wellbeing. More than one participant risked health consequences from DNP simply for satisfying curiosity (e.g., *“want to see what it did, I was on it for a week only”* and *“to see if it actually worked that good*”).

One participant, who paradoxically expressed very high level of concern about DNP but had previous experience with illegal supplements, said: *“[I expected] moderate fat loss, bloating, a lot of sweating, possibly falling over and dying but meh…”*. Another participant, who expressed no concern about DNP stated on the question how it was ensured that the DNP purchased was genuine: *“test[ed] it in my body and see what happens”*. Others were more cautious and thoroughly researched DNP before they decided to try it. For example, a 22 year old male said: *“To be honest I was rather scared, once I did a little 10 day cycle I was comfortable with it. I’m on my third cycle right now. Started at 261lbs. Take all the supplements and don’t be an overzealous fat retard and you’ll do fine on DNP.”* Another participant, 45 years old male, said: *“I did a lot of research, exercised a lot of caution, and have had a life benefitting experience with DNP. My research led me to see that most who died from DNP had either eating disorders or a lack of self control/and poor judgement. Death is hardly the norm.”* In both responses, the prevailing belief among DNP users manifests, namely that those who were in serious trouble with or died because of DNP either made a mistake (considered as an avoidable factor) or suffered from an eating disorder (considered as a non-relevant feature).

#### Normative perceptions

**Fig. 5:**
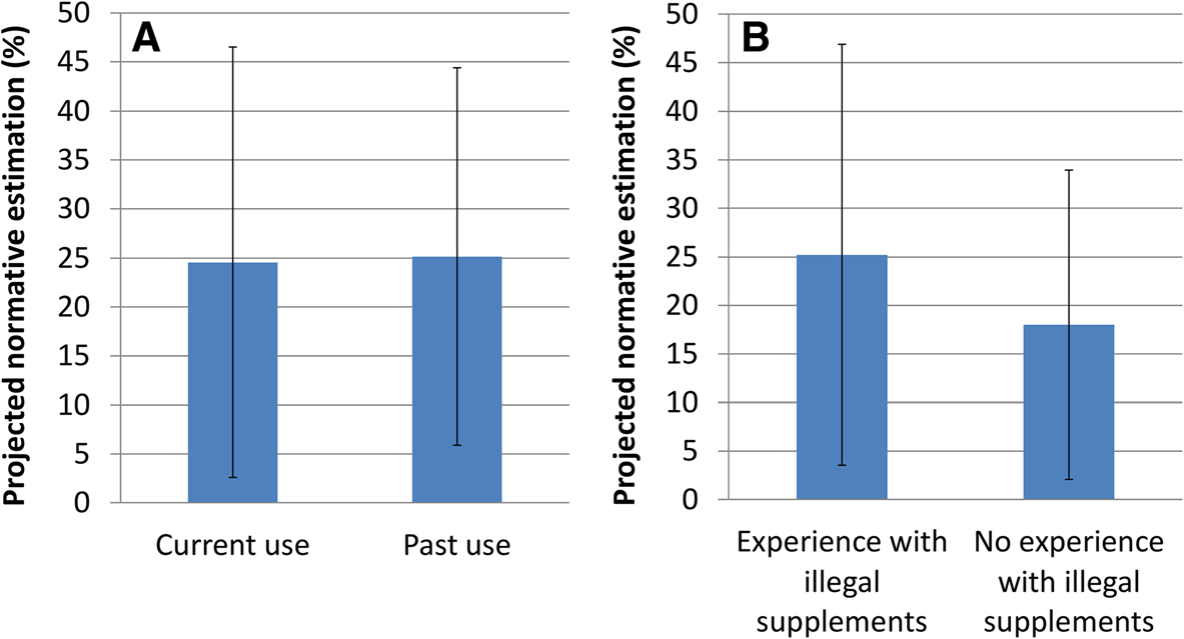
Comparison of the perceived normative estimation of DNP use among bodybuilders and avid exercisers based on (**a**) whether fat-burner substance is used at the time of data collection and (**b**) having experience with other illegal supplements

On average, DNP users estimated that a quarter of bodybuilders (23.05 ± 20.00 %) use DNP. Detailed normative perceptions by user details (current use of fat-burner and having experience with other illegal supplements) are depicted in Fig. 5. The difference between current fat-burner users and non-users was small and non-significant (*t*(16) = 0.059, *p* = 0.954, *d* = 0.029, 24.55 ± 21.96 % vs. 25.14 ± 19.25 % for current users (*n* = 11) and non-users (*n* = 7) respectively). The small effect size suggests that current behaviour with similar drugs does not influence the estimation regarding DNP. Equally, there was an observed but statistically not significant difference in estimations (*t*(18) = 0.730, *p* = 0.478, *d* = 0.379) made by those who only used DNP (*n* = 6; mean estimation = 18.00 ± 15.94 %) from the array of ‘illegal supplements’ versus those with more experience (*n* = 14; mean estimation = 25.20 ± 21.68 %). From literature precedence, it was expected that the perceived legality of the drug - possibly through mental representation of DNP - could influence the estimation of DNP use among others. In similar context, it has been observed that athletes involved in socially detested behaviour (e.g., doping) tend to overestimate the same behaviour among others compared to those who are absent, but with no difference in estimations of the use of performance enhancing aids such as nutritional supplements that can be used without restrictions [67–69]. In the present sample, the observed difference (18 % vs. 25 %) lacks statistical support for the legality effect on projected use; and thus may only reflect random variation in the sample. Future research specifically tailored to investigate projection bias is needed to ascertain if the difference manifests in subsequent studies with robust evidence for statistical significance.

DNP represents a grey area on multiple accounts. Technically, DNP is not an illegal drug to take, but it is not licensed for human consumption, thus selling for such purpose is illegal. From the cognitive point of view, it is an interesting case because DNP may very well be perceived as illegal owing to the clandestine nature of purchase and use, even if from the legislative point of view, there is nothing illegal about buying and using DNP. A similar phenomenon has been observed regarding performance enhancing nutritional supplements among athletes [70]. Thus we expected that those using other illegal substances may automatically class DNP as illegal whereas those who do not use anything illegal had a more accurate view. Perhaps it is because legality is important to these users, not wanting to cross the border between legal and illegal; whereas those with experience with illegal supplements already did so. Further research with experimental design manipulating the legality condition is needed to ascertain whether a heuristic bias regarding DNP exists (i.e., DNP users who also use other illegal bodybuilding substances may subconsciously include DNP in that category whereas those who stay away from illegal bodybuilding substances may not see DNP as illegal). The importance of this aspect lies in the potential effect on prevention strategies and communication.

#### Contrast between the Internet and real life

**Fig. 6:**
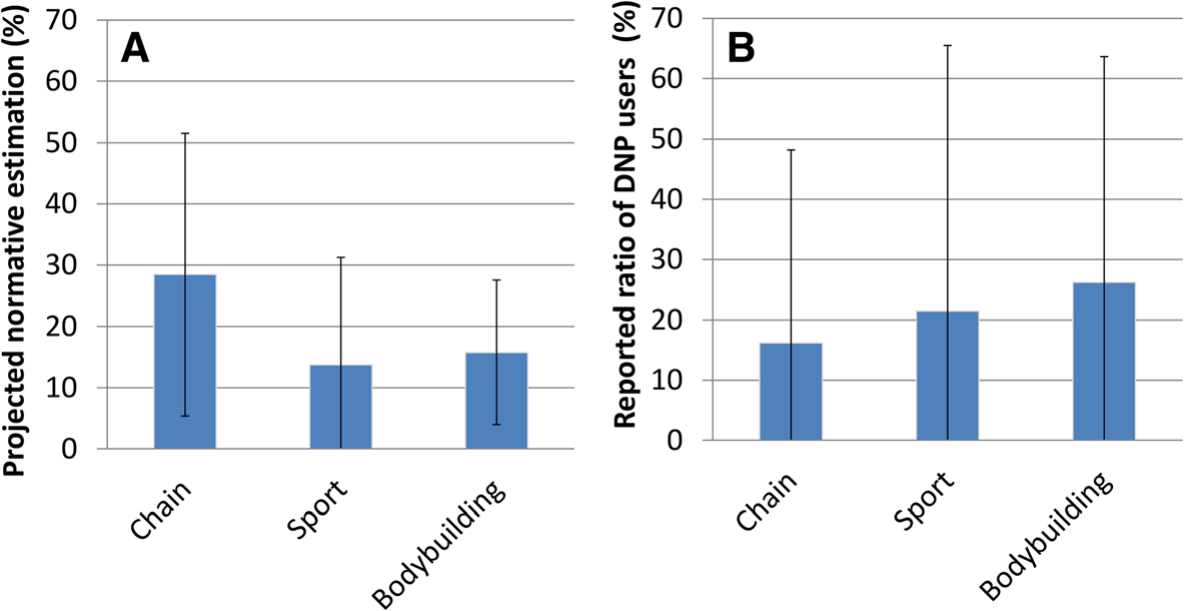
Comparison of (**a**) the perceived normative estimation of DNP use among bodybuilders and avid exercisers and (**b**) visibility of DNP use by the type of gym used in the sample

Break-down of the estimations by the type of gym (Fig. 6a) shows an interesting - and intuitively unexpected -pattern. DNP users attending leisure/exercise-focused ‘chain’ gyms give much higher estimation for DNP use among others (28.45 ± 23.09, *n* = 11) than those attending sport- or bodybuilding focused gyms (13.75 ± 17.50; *n* = 4 and 15.75 ± 11.79; *n* = 4), respectively). Although the difference in projected estimations did not reach statistical significance, the effect size suggests that the lack of statistical significance is the function of the interplay between the small sample and large variance (*F*(2,19) = 1.053; *p* = 0.372; *η*^*2*^ = 0.116), resulting in the test being underpowered to reject the null hypothesis when the difference between the three groups may exist. Further research with adequately powered statistical test is warranted.

On the contrary, the visibility of DNP use through the reported known proportion of DNP users (Fig. 6b) shows the opposite pattern, with the highest proportion reported by people attending bodybuilding focused gyms (0.262 ± 0.375 vs. 0.215 ± 0.440 vs. 0.162 ± 0.320, respectively for bodybuilding (*n* = 6), sport (*n* = 5) and leisure/exercise-focused (*n* = 10) gyms). The difference was not statistically significant and the effect size was small (*F*(2,18) = 0.144, *p* = 0.867, *η*^*2*^ = 0.016). The reported proportion of known DNP users was surprisingly low across the sample. Against the average number of bodybuilders personally known by the sample (11.38 ± 14.50), and the estimated normative prevalence at 23 %, the known number of DNP users among them (1.12 ± 2.05) barely reaches 10 %. The average number of DNP users expected from the projected prevalence (2.63 ± 4.07) significantly exceeded the average number (1.24 ± 2.46) of reported known users (Wilcoxon *Z* = −2.274, two-tailed exact *p* = 0.021, point probability *p* = 0.002), with a significant positive correlation between the reported and estimated DNP user numbers (Spearman *r*(18) = 0.665, *p* = 0.003).

Taken together, these results suggest that DNP practices and experiences are shared online, but not in real life. DNP use appears to take place in isolation. This may partly so because of the social disapproval of DNP use, even among bodybuilders; but also because managing the side effects (profuse sweating, skin discoloration, etc.) in public is difficult. In fact, new users are advised in discussion forums to start the cycle when on holiday if they are in employment. Conceivably, sharing experiences online is a way to deal with isolation and loneliness during a DNP course. If this is the case, online forums play an important role beyond being a compendium of DNP knowledge; and it warrants further investigation.

During data collection we encountered numerous cases where people, who willingly shared their experiences with DNP and readily offered information and advice to other forum users, were initially suspicious of a newcomer asking questions about DNP and were not very willing to participate until the investigator gained trust and became accepted in this unique group. Perhaps DNP in a sporting context, owing to its high risk and harsh side effects, is similar to harsh drugs among substance users, where people are much more ready to admit using a relatively harmless substance (e.g., smoking marijuana) than confessing a more stigmatising “hard drug” use (e.g., snorting cocaine or injecting heroin). Whilst the stigma attached to psychoactive drugs has two facets (addiction and criminalisation), for DNP, it is conceivable that such distinction is mainly driven by the degree of health risk and rather than legal consequences.

The high estimating of use by leisure/exercise gym users (which is likely to be an over-estimation at 28 %) is concerning on two counts. First, against the relatively low reported number of deaths from DNP, it can create a false sense of security by believing that DNP use is a common and safe practice. Secondly, this group is likely to be the most naive and least experienced when it comes to using “hard” sport drugs (e.g., anabolic steroids, hormones, other fat-burners such as Clenbuterol), dosing properly and managing adverse side effects. As one ex-professional powerlifter (a 22 year old male) offered for explanation: “*Some bodybuilders may use other drugs to the same effect- clen [clenbuterol] is especially popular, that is why my estimate for DNP use is so low”*. Another participant, a 23 year old male, linked DNP use to anabolic steroids: *“Typically DNP users are mostly bodybuilders that are already on anabolics. I was not on anabolics and I have never taken them so my results may not have been as good as others on online forums.”.* Because we did not ask the participants to reveal full details of their supplement and substance use, future research is required to ascertain the relationship between DNP and other, legal and illegal, substances.

## General discussion and policy recommendations

It is reassuring that pure DNP (or as active ingredient at the typical dose of 200 mg) was not found among the supplement samples. However against this background, a potentially dangerous discrepancy emerged between the views of users and regulators about how DNP is marketed and sold. Whilst DNP is marketed as an effective weight-loss aid, DNP as active ingredient is disguised for shipping purposes only (i.e., labelled as turmeric; or shipped in unlabelled ‘discreet packaging’ to avoid detection and confiscation by customs or the police) but it is clear to the users what they ingest. Owing to the grey area in legislation, there is no need for sellers to disguise DNP under fictitious supplement names to evade FSA or equivalent regulatory bodies worldwide. DNP can be, and is sold ‘as is’ over the Internet, as long as it is labelled as an industrial- or research chemical. On one hand, it is comforting because it lessens the chance for inadvertent exposure. Paradoxical as it might be, it is also in the sellers’ best interest to supply DNP with a formal warning that it is not intended for human consumption because it shifts the responsibility from the seller to the user. The user who knowingly ingests DNP despite the legal and health warnings, for all intents and purposes, makes an informed choice and willingly and voluntarily put his/her own health at risk. If DNP is - hypothetically - sold in disguise as a fat-burned supplement without specific reference to DNP being the active ingredient, if something goes wrong, the manufacturer/seller can be charged with manslaughter. On the other hand, with positive feedback and supportive attitude toward DNP use as an effective fat-burner brimming the Internet, coupled with the easy availability of DNP, there is a danger that naive dieters or members of vulnerable groups who are unable to make rational and informed decisions about weight-management practices, are tempted to try DNP. Because the potential DNP user groups are qualitatively different, a one fits all approach to prevention is not likely to be sufficient. The naive and vulnerable need protection with strong preventive measures, whereas the community of determined and committed DNP users would benefit from information, medical help and harm-reduction. Regulators and policy makers are advised to proceed with care in this tightrope-balance situation. Tightened access control is likely to protect the general public but will not dissuade determined users from DNP. As it has been observed with other illegal- and performance-enhancing substances [71, 72], if selling the substance poses too high of a risk to distributors, social dealers who are socioculturally similar to buyers move out and are replaced by organised crime. It has been established that increase in punishment for the lesser crime inevitably drives criminal activities toward the more serious end of the spectrum if the more serious crime also means a reduced chance of being caught [73].

Because of the importance of trust, reputation and personal relationship, it is further assumed that the anonymity of the ‘dark net’ does suit the bodybuilding drug market. Based on one dominant outcome of this study, namely how DNP users ensure quality and safety, it is reasonable to assume that relationship between buyers and suppliers in the bodybuilding drug market resembles a form of social dealership; albeit in most cases, the relationship maybe virtual and transactions are made through the means of the Internet. Thus, trust in suppliers may have different dimensions which are possibly negotiated through additional roles (e.g., also supplying advice about what substance and how to use, including harm-reduction measures or selling other, more commonly used performance enhancers). If this hypothesis gains empirical support in the future, then suppliers indeed play an important role in keeping the bodybuilding drug market relatively safe. If harsher penalties drive the knowledgeable, and somewhat customer-oriented suppliers out of the market because selling such substances is no longer worth the risk, then they will inevitably be replaced by the profit-oriented suppliers without knowledge specific to the drugs (e.g., anabolic steroids, fat-burners, pre-workout stimulants) or without understanding of their clients. Then DNP may move out from the highly visible Internet to the invisible dark net, making it less traceable or controllable. It may also motivate manufacturers to market DNP in disguise.

Contamination or adulteration with DNP was found in 14.3 % of the tested bodybuilding supplements in low concentration. Almost half (44.4 %) of all Internet-sourced samples were contaminated compared to only 7.7 % of those obtained from high street retailers, which suggests that controlling effort is best concentrated on supplements available - or in some cases, only available - from online retailers. The low representation of gym sample prevents making meaningful conclusion but future investigations should specifically focus on samples bought in gyms because the original source of these supplements could be the generally cheaper online retail network and/or underground labs. The concentration of DNP in these supplements was well below the levels of use which bodybuilders report, and far from the significant exposure of 30 mg/kg dose used in an *in vivo* rat study to induce sufficient increase in energy demand for fat loss [74]. Thus, deliberate adulteration and violation of the labelling requirement [38] is less likely than contamination owing to poor quality control.

Despite warnings issued by food standard agencies and national health services, online retail sites that are actively promoting and selling DNP are easily found on the Internet. In online retail sites, information on health risks and side effects associated with DNP use are not always listed but when they are, their seriousness is downplayed and advice is offered for avoiding side effects and “safe” use. People who are interested in trying DNP can find a plethora of accounts of first hand experiences and user advice on how to avoid side effects including dosage, duration, incremental increase in amount taken to build tolerance, recommended daily water intake and diet plans to follow while taking DNP from discussion forums and blogs.

The survey results, coupled with the Internet forums, provided immutable evidence that DNP is knowingly used whilst the risks associated with such use is acknowledged. Motivated by the desirable goal (fat loss, either as an end result or a mean to an end), dedicated bodybuilders and exercisers have used and, by large, plan to continue using DNP. This group appears to be qualitatively different from those described in the media for DNP death- or near-death incidents. DNP users in our sample specifically sought DNP with the intention to use or try; as opposed to using a slimming aid that happens to be or contains high dose of DNP.

Based on the quantitative results and qualitative accounts, DNP users’ profiles contradict the commonly held views of “mindless risk-takers” and appear to be very similar to the profiles observed for non-medical steroid users [75]. Their approach to DNP focuses on being experienced in controlling weight and in controlled weight-loss practices, and they warn new users against thinking of DNP as a quick fix. In fact, DNP users exhibited a high degree of control and knowledge as well as exercised precautions. Experienced DNP users act as gatekeepers for sensible and safe use, which indicates the presence of a grass-root, self-organised and sustained harm-reduction. This observation is underscored by the overwhelmingly strong theme that emerged from the qualitative responses, expressing the desire for accurate knowledge about DNP and help in using DNP safely. Notably the language used in qualitative responses is remarkably similar to those shown in studies investigating bodybuilders’ views about anabolic steroids and other performance enhancing substances [76–81]. However, interpreting these as evidence for moral disengagement [82] may inadvertently pose a moral/ethical frame to a behaviour which – at least at the time of this study in this population - does not break legal or social rules. It is without doubt that DNP use is a controversial behaviour, even among bodybuilders, and highly risky in terms of potential health consequences. Applying the moral disengagement process of cognitive restructuring of inhumane conduct against others into a worthy or acceptable behaviour inevitably labels DNP use as ‘inhumane’ or ‘immoral’. Apart from the crucial difference that DNP directly affects no one but the user (i.e., there is no victim), this is the precise labelling which DNP users argue against. Instead, in the current legislative context, downplaying these health consequences and making advantageous comparisons, coupled with expressing high confidence in control over use, is better interpreted as rationalisation mechanism typically employed in functional use of mind-altering drugs [40]. As part of the normalisation process, rationalising using performance enhancing drugs with similar arguments has been evidenced previously [83–86]. Although both interpretations have academic merits and are valuable from the theoretical point of view, the functional interpretation is able to provide more direct and practically relevant avenues for prevention and/or harm-reduction than the moral/ethical argument.

The clear evidence from Internet forums for use and people’s willingness to use ‘uncut’ DNP calls for an urgent shift in preventive actions. Instead of efforts targeting supplements and detecting contamination, the concentrated focus should be on sales of ‘uncut’ DNP over the Internet whilst addressing the public health concern in a more convincing way. Preventive efforts would further benefit from research into the most effective message framing and delivery to discourage people from risky experimentation with unknown and unlicensed body-image enhancing substances.

Accepting that some people will not be deterred from self-experimenting with effective appearance and performance-enhancing drugs (such as DNP) ways of harm-reduction should be considered. In the absence of authoritative information on “safe use” of DNP, mainly because such advice cannot be confidently given, online discussion boards serve as sources of knowledge on DNP. This is similar to the role the Internet plays in the current drug culture [87] and related knowledge exchange [88, 89]. Although Internet forums about DNP are generally supportive of intended users in terms of sharing experiences and advising them on “safe use”, the particular danger with DNP lies in the significant inter-individual differences in the presence and severity of the toxic effects; as well as in the very narrow window between effective and toxic dose. Unlike with most novel psychoactive substances, where harm can most likely to be mitigated via advice on safe use, the combination of DNP dosage and length of the cycle that works safely and effectively for one may kill or seriously harm another. A harm reduction approach via providing accurate and scientifically evidenced information highlighting the lack of established safe levels for DNP could effectively prevent naive users playing Russian roulette with the substance. In the absence of this, the only information source at the disposal of the interested users is the Internet and where they can only turn to forums and blogs for advice.

## Limitations and directions for future research

The study, being the first of this type on DNP, inherently carries several limitations. The multi-study nature of this project did not afford more in-depth exploration of one particular aspect but opened interesting avenues and raised important questions for future research. In the effort to keep the survey as succinct as possible, details on concomitant supplement use and experience with other ‘illegal supplements’ were not sought. A further potential limitation here is that specific definition for ‘illegal supplements’ was not given to the participants, thus responses to this question are influenced by the individual interpretations what constitute illegal; and such interpretation might had a carry-over effect on the prevalence perception of DNP use.

Another limitation of this study is the small sample size from gym donations for testing, which prevents us drawing meaningful conclusions for the general population of recreational or fitness exercisers. The relatively small sample of DNP users renders testing for statistically significant differences difficult and results arising from statistical analyses should be interpreted cautiously. They are presented to illustrate possible trends to inform future research directions rather than drawing definite conclusions. For the latter, the research should be repeated in a larger sample. However, it must be noted that although by the general standards of survey methodology, the sample size may appear small; owing to the high level of suspicion that surrounds any enquiry about DNP by a newcomer who is not known in the online discussion board community, recruiting 35 users is a considerable achievement which took approximately nine months. With the small sample size limitation for quantitative analysis acknowledged, the open-ended questions however yielded rich qualitative information from a significant cohort of DNP users on how DNP users feel about DNP, rationalise their behavioural choice and manage the risks that they are fully aware of.

Future studies should address the limitations of this study whilst moving the research forward toward a better understanding of the complexity of behavioural motivations and reasons in these distinctively different populations of DNP users. Studies focusing on people with disordered eating and extreme dieters will make valuable contributions, so would expanding the scope of personality factors and situational demands. Future investigation could also focus on the cognitive process of dealing with conflicting information (i.e., DNP is very dangerous vs. DNP can be used safely with care) in the context of weight-related goals and past experiences, along with associated risk perception, compensatory belief mechanism (particularly in the light of the elevated risk willingness if past experience with similar drugs are available) and trust in information sources and purchase options. Findings from these lines of enquiry may then be further expanded and extrapolated to other substance categories such as stimulants, nootropics and novel psychoactive substances (legal highs). It is hypothesised that the customer side of the DNP market might be similar to users of “legal highs” where the market is not homogeneous in terms of knowledge and motivation. Distinguishing between user groups could be vital for targeted prevention and harm reduction. Because the majority of the DNP users reported concomitant supplement use, further investigation is warranted to explore what other substances are used while on DNP regime. Future investigations should also explore if side effects or severity of side effects are associated with a specific form in which DNP was purchased. This could be an important factor for harm-reduction but the present study does not have sufficiently detailed information to adequately test this hypothesis.

Our results also indicated that DNP users’ trust in both the substance and in the quality and validity of the information on the substance is largely based on reputation in the source. Public health authorities and regulatory bodies would benefit from a better understanding of the role of trust in information in situations that carry a degree of health-risks and transgresses social and legal boundaries.

Although it could be argued that the majority of the users in the present sample may not have a real need for the desired fat loss, reporting already low body fat percentage, DNP use is not limited to this particular population. Considerable increases in the prevalence of obesity and diabetes flag these as global health challenges which warrant high levels of focused research. DNP has undoubtedly a major contribution to make for weight loss, should the side-effect profile be controlled. A recent report reveals that controlled-release formulation of DNP had considerable health benefits including reversing diabetes but with no toxicity in rat models [90]. Further expansion into modern formulations involving slow-release and patch-delivery, alongside pre-dose sensitivity tests, could place DNP within the clinicians’ arsenal to combat obesity and some forms of diabetes; and may reduce harm by offering a safer alternative to misusing bulk industrial formulations or underground products.

That is not to promote a *laissez fair* attitude toward DNP, or support or encourage unlicensed substance use. Quite the contrary, we propagate a pragmatic view to facilitate the development of evidence-based, effective and ecologically valid prevention- and harm-reduction strategies. A recent report by Advisory Council on the Misuse of Drugs (ACMD) clearly showed that successful efforts to drug- and substance use prevention are likely to be multi-sectoral with multiple components [91]. Keeping DNP use (along with similar substances) under control is feasible if legislation, law enforcement, health-care, public health social marketing campaigns, authorities of food- and drug safety and education work in concert. Environmental prevention through market and marketing controls should work in tandem with social marketing delivering balanced and accurate information and health-care support, including offering alternatives to address the driving forces behind DNP use and engage in harm-reduction where prevention is not achievable. Stand-alone social marketing campaigns focusing on illegality and/or danger in general terms should be avoided as they are likely to be, at best, ineffective and may even lead to increased interest and use. Striking the right balance between restriction of access; legal consequences of producing, selling and using; research advances into safety and psychosocial interventions through education which target the underlying reasons and motives could form positive synergy for a holistic approach and effectively address this public health concern in a pragmatic way.

## Conclusion

Contamination or adulteration with DNP may violate labelling and manufacturing requirements for dietary supplements but accidental ingestion, owing to the low level found, does not appear to pose significant health risks to the public. Public health concern however is linked to the deliberate use and willingness to use uncut compound despite the health warning and the lack of control that surrounds the quality and availability of this highly dangerous substance to inexperienced or naive users.

To mitigate health risks people willingly take with unknown, unlicensed and potentially dangerous substances is to devise end-user-centred, proactive public health policies. For the first time in research on DNP, this study gave voice to the users and presents their authentic view in a non-judgemental way to inform public health policies. Off-label use of DNP is unlikely to cease and thus realistic attempts should be made to address the issues. Controlling efforts should focus on ‘uncut’ DNP and the Internet. In order to protect the experienced and the vulnerable (inexperienced, naïve and those suffering from eating disorders or some other relevant psychological disorders), it is important to provide accurate and trustworthy information; and acknowledge that scare tactics may not work when ‘positive’ accounts are readily available on the Internet. The most critical point that social marketing information should stress is the lack of established safe limit. Owing to the wide variation in individual sensitivity to DNP, what works for most or many - as detailed on the Internet forums and blogs - could be toxic or even lethal to others. For the first time, it is a Russian roulette for every single user.

Effective prevention should even move beyond knowledge-based intervention (such as issuing health warnings regarding DNP) and simultaneously tackle the motivations and tangible reasons behind the potentially risky behaviour with unknown or unlicensed drugs and incorporate harm-reduction measures as well as making investment into researching a safer but equally potent formulation. Users’ accounts leave no doubt that DNP use can only be curbed if comparable alternatives are available.

## Additional files

Additional file 1:
**Method for the semi-quantitative screening for 2,4 dinitrophenol (DNP).** (PDF 190 kb) Additional file 2:
**List of retail web-based sites and discussion boards/forums.** (PDF 389 kb)

## References

[1] James WPT (2008). The epidemiology of obesity: the size of the problem. J Internal Med.

[2] Müller-Riemenschneider F, Reinhold T, Berghöfer A, Willich SN (2008). Health-economic burden of obesity in Europe. Eur J Epidemiol.

[3] Ford ES, Mokdad AH (2008). Epidemiology of obesity in the Western Hemisphere. J Clin Endocrinol Metabol.

[4] Yuliana ND, Jahangir M, Korthout H, Choi YH, Kim HK, Verpoorte R (2011). Comprehensive review on herbal medicine for energy intake suppression. Obes Rev.

[5] Kim HJ, Lee JH, Park HJ, Cho SH, Cho S, Kim WS (2014). Monitoring of 29 weight loss compounds in foods and dietary supplements by LC-MS/MS. Food Addit Contam Part A Chem Anal Control Expo Risk Assess.

[6] Monakhova YB, Kuballa T, Löbell‐Behrends S, Hengen J, Maixner S, Kohl‐Himmelseher M (2012). 1H NMR screening of pharmacologically active substances in weight‐loss supplements being sold online. Lebensmittelchemie.

[7] Rebiere H, Guinot P, Civade C, Bonnet PA, Nicolas A (2012). Detection of hazardous weight-loss substances in adulterated slimming formulations using ultra-high-pressure liquid chromatography with diode-array detection. Food Addit Contam Part A Chem Anal Control Expo Risk Assess.

[8] Lachenmeier DW, Löbell-Behrends S, Böse W, Marx G (2013). Does European Union food policy privilege the internet market? Suggestions for a specialized regulatory framework. Food Control.

[9] Cohen PA, Goday A, Swann JP (2012). The return of rainbow diet pills. Am J Public Health.

[10] Yen M, Ewald MB (2012). Toxicity of weight loss agents. J Med Toxicol.

[11] Miranda EJ, McIntyre IM, Parker DR, Gary RD, Logan BK (2006). Two deaths attributed to the use of 2,4 dinitrophenol. J Anal Toxicol.

[12] Grundlingh J, Dargan PI, El-Zanfaly M, Wood DM (2011). 2,4-Dinitrophenol (DNP): A weight loss agent with significant acute toxicity and risk of death. J Med Toxicol.

[13] Kamour A, George N, Gwynnette D, Cooper G, Lupton D, Eddleston M, et al. Increasing frequency of severe clinical toxicity after use of 2, 4-dinitrophenol in the UK: a report from the National Poisons Information Service. Emerg Med J. 2014;doi:10.1136/emermed-2013-203335. 10.1136/emermed-2013-203335 24957806

[14] Lee HCH, Law CY, Chen ML, Lam YH, Chan AYW, Mak TWL (2014). 2, 4-Dinitrophenol: A threat to Chinese body-conscious groups. J Chin Med Assoc.

[15] Food Standards Agency. Warning about ‘fat-burner’ substances containing DNP. http://www.food.gov.uk/news-updates/news/2012/5371/dnp-warning. Accessed on 06 April 2015.

[16] Food Standards Agency. Help prevent another DNP death. http://www.food.gov.uk/news-updates/news/2013/5833/dnp. Accessed on 06 April 2015.

[17] Wise J (2014). Increase in reports of toxicity from fat burning supplement seen in UK. BMJ.

[18] Lemon TI (2013). 2, 4 Dinitrophenol - a danger for patients with drug use issues and eating disorders alike. J Sports Med Doping Stud.

[19] Petróczi A, Naughton DP (2010). Potentially fatal new trend in performance enhancement: a cautionary note on nitrite. J Int Soc Sports Nutr.

[20] Pomeranz JL, Barbosa G, Killian C, Austin SB. The dangerous mix of adolescents and dietary supplements for weight loss and muscle building: legal strategies for state action. J Public Health Manag Pract. 2014; doi:10.1097/PHH.0000000000000142. 10.1097/PHH.0000000000000142 25248073

[21] Harper JA, Dickinson K, Brand MD (2001). Mitochondrial uncoupling as a target for drug development for the treatment of obesity. Obes Rev.

[22] Le P, Wood B, Kumarasinghe SP. Cutaneous drug toxicity from 2, 4‐dinitrophenol (DNP): Case report and histological description. Australas J Dermatol. 2014; doi:10.1111/ajd.12237. 10.1111/ajd.12237 25367505

[23] Reid SL, Salmond J, Datta S, Underwood MA, Akhtar MN. Dietary supplement 2, 4-dinitrophenol resulting in Fournier’s gangrene. JCU. 2014; doi: 10.1177/2051415814551379.

[24] Ravesloot JH, Rombouts E (2000). 2,4-Dinitrophenol acutely inhibits rabbit atrial Ca^2+^ -sensitive Cl^−^ current (I_TO2_). Can J Physiol Pharm.

[25] Wu SN, Li HF, Chiang HT (2000). Characterization of ATP-sensitive potassium channels functionally expressed pituitary cells. J Membr Biol.

[26] Agency for Toxic Substances & Disease Registry. Public health statement. dinitrophenols. CAS ID: 51-28-5 (2,4-DNP). 1995. http://www.atsdr.cdc.gov/ToxProfiles/tp64-c1-b.pdf. Accessed on 06 April 2015.

[27] National Health Service. New warnings issued over deadly DNP ‘diet drug’. http://www.nhs.uk/news/2014/01January/Pages/New-warnings-issued-over-deadly-DNP-diet-drug.aspx. Issued on 15/01/2014. Accessed on 06 April 2015.

[28] National Health Service. Warnings issued over deadly DNP diet drug. http://www.nhs.uk/news/2013/09September/Pages/Warnings-issued-over-deadly-DNP-diet-drug.aspx Accessed on 06 April 2015.

[29] FSA action over DNP ‘fat burner substances’. http://www.food.gov.uk/news-updates/news/2013/5772/dnp. Accessed on 06 April 2015.

[30] Risks of buying medicines over the Internet. http://www.nidirect.gov.uk/risks-of-buying-medicines-over-the-internet. Accessed on 11 October 2015.

[31] Montoya ID, Jano E (2007). Online pharmacies: safety and regulatory considerations. Int J Health Serv.

[32] Rosenbaum CD, Carreiro SP, Babu KM (2012). Here today, gone tomorrow… and back again? A review of herbal marijuana alternatives (K2, Spice), synthetic cathinones (bath salts), kratom, Salvia divinorum, methoxetamine, and piperazines. J Med Toxicol.

[33] Schmidt MM, Sharma A, Schifano F, Feinmann C (2011). “Legal highs” on the net—Evaluation of UK-based Websites, products and product information. Forensic Sci Int.

[34] Hoxha B, Petróczi A (2015). Playing with fire? Factors influencing risk willingness with unlicensed fat burner drug 2,4-Dinitrophenol (DNP) in young adults. Publ Health.

[35] Redlich-Amirav D, Higginbottom G (2014). New emerging technologies in qualitative research. Qual Report.

[36] Holtz P, Kronberger N, Wagner W (2012). Analyzing internet forums. A practical guide. J Media Psychol – GER.

[37] Im EO, Chee W (2012). Practical guidelines for qualitative research using online forums. Comput Inform Nurs.

[38] Number 19 of the Food Labelling Regulations 1996, as amended by the Food Labelling (Amendment) Regulations 1998 and the Food Labelling (Amendment) (No. 2) Regulations 1999. The National Archives [http://www.legislation.gov.uk/uksi/1996/1499/contents/made]

[39] Westaby JD (2005). Behavioural reasoning theory: identifying new linkages underlying intentions and behaviour. Organ Behav Hum.

[40] Müller CP, Schumann G (2011). Drugs as instruments: A new framework for non-addictive psychoactive drug use. Behav Brain Sci.

[41] Marteau TM, Dormandy E, Mitchie S (2001). A measure of informed choice. Health Expect.

[42] Fritz CO, Morris PE, Richler JJ (2012). Effect size estimates: current use, calculations, and interpretation. J Experiment Psychol Gen.

[43] Richardson JTE (2011). Eta squared and partial eta squared as measures of effect size in educational research. Educational Res Rev.

[44] Cohen J (1988). Statistical power analysis for behavioural sciences (2^nd^ Ed.).

[45] Johnson VE (2013). Revised standards for statistical evidence. Proc Natl Acad Sci U S A.

[46] Lipsey MW, Wilson D. Practical meta-analysis. Applied social research methods. (1^st^ Ed.). Thousand Oaks:Sage 2001. http://www.campbellcollaboration.org/escalc/html/EffectSizeCalculator-R5.php.

[47] Guest G, MacQueen KM, Namey EE. Applied thematic analysis. Thousand Oaks:Sage

[48] Braun V, Clarke V, Terry G. Thematic analysis. In P Rohleder, EC Lyons Editors. Qualitative Research in Clinical and Health Psychology, 2015; 95–113. New York: Palgrave Macmillan.

[49] King RA, Racherla P, Bush VD (2014). What we know and don’t know about online word-of-mouth: A review and synthesis of the literature. J Interact Marketing.

[50] Utz S, Kerkhof P, van den Bos J (2012). Consumers rule: How consumer reviews influence trustworthiness of online stores. Electronic Commerce Res Appl.

[51] Eastlick MA, Lotz S (2011). Cognitive and institutional predictors of initial trust toward an online retailer. Int J Retail Distrib Management.

[52] Beldad A, de Jong M, Steehouder M (2010). How shall I trust the faceless and the intangible? A literature review on the antecedents of online trust. Comp Human Behav.

[53] Davey Z, Schifano F, Corazza O, Deluca P (2012). e-Psychonauts: conducting research in online drug forum communities. J Mental Health.

[54] Orsolini L, Duccio Papanti G, Francesconi G, Schifano F (2015). Mind navigators of chemicals’ experimenters? A web-based description of E-psychonauts. Cyberpsychol, Behav Social Networking.

[55] Miller S-J. Healthy weight & height for bodybuilding. 2014. http://www.livestrong.com/article/372555-healthy-weight-height-for-bodybuilding/. Accessed on 06 April 2015.

[56] Moody A. Adult anthropometric measures, overweight and obesity. In Craig R, Mindell J, editors. Health Survey for England – 2012 (Report). Volume 1: Health, social care and lifestyles. Health and Social Care Information Centre. 2013. http://www.hscic.gov.uk/catalogue/PUB13218/HSE2012-Ch10-Adult-BMI.pdf

[57] Jansen KL, Prast CJ (1988). Psychoactive properties of mitragynine (kratom). J Psychoactive Drug.

[58] Sanghani R. ‘I know the dangers but I want them anyway’: The dark truth about diet pills. The Telegraph;April 21^st^, 2015. http://www.telegraph.co.uk/women/womens-health/11552359/Diet-pills-I-know-the-danger-but-I-want-them-anyway.html

[59] Choi C. Heart problems and heroin: Welcome to the world of slimming pills. ITV News; July 23^rd^. 2015. http://www.itv.com/news/2015-07-23/heart-problems-and-heroin-welcome-to-the-world-of-slimming-pills/

[60] Neumark-Sztainer D, Wall M, Larson NI, Eisenberg ME, Loth K (2011). Dieting and disordered eating behaviors from adolescence to young adulthood: findings from a 10-year longitudinal study. J Am Dietetic Assoc.

[61] Haedt AA, Keel PK (2010). Comparing definitions of purging disorder on point prevalence and associations with external validators. Int J Eating Disorder.

[62] Reba-Harrelson L, Von Holle A, Thornton LM, Klump KL, Berrettini WH, Brandt H (2008). Features associated with diet pill use in individuals with eating disorders. Eating Behav.

[63] Ratcliff R. Diet pills website ‘should have been shut down’ a year before student’s death. The Guardian; July 25^th^ 2015. http://www.theguardian.com/uk-news/2015/jul/25/diet-pills-website-parry-student-death

[64] Rawstorne T. Killed by the tablets they took to lose weight: This beautiful student doctor was killed by internet slimming pills that make users fatally overheat. And she is far from the only victim. The Mail on Sunday; 26 April 2013. http://www.dailymail.co.uk/femail/article-2315433/Sarah-Houston-cause-death-Boiled-alive-internet-slimming-pills-DNP.html

[65] Man killed by herbicide ‘weight loss aid’, court hears. The Guardian; July 8^th^, 2014. http://www.theguardian.com/uk-news/2014/jul/08/man-killed-herbicide-weight-loss-aid-court-hears

[66] Man dies ‘after taking diet pills’. Independent.ie; 25^th^ June, 2015. http://www.independent.ie/breaking-news/irish-news/man-dies-after-taking-diet-pills-31330421.html

[67] Petróczi A, Mazanov J, Naughton DP (2011). Inside athletes’ minds: Preliminary results from a pilot study on mental representation of doping and implications for anti-doping. Subst Abuse Treat Prev Policy.

[68] Uvacsek M, Ránky M, Nepusz T, Naughton DP, Mazanov J, Petroczi A (2011). Self-admitted behaviour and perceived use of performance enhancing versus psychoactive drugs among competitive athletes. Scand J Med Sci Sports.

[69] Petroczi A (2013). The doping mindset - Part I: Implications of the functional use theory on mental representations of doping. PEH.

[70] Petróczi A, Mazanov J, Naughton DP (2011). Inside athletes’ minds: Preliminary results from a pilot study on mental representation of doping and potential implications for anti-doping. Subst Abuse Treatment Prev Policy.

[71] Fincoeur B, Frenger M, Pitsch W (2013). Does one play with the athletes’ health in the name of ethics?. PEH.

[72] Fincoeur B, Van de Ven K, Mulrooney KJ. The symbiotic evolution of anti-doping and supply chains of doping substances: how criminal networks may benefit from anti-doping policy. Trends Organ Crime. 2014; doi:10.1007/s12117-014-9235-7.

[73] Becker GS, Becker GS, Landes WM (1974). Crime and punishment: An economic approach. Essays in the economics of crime and punishment.

[74] Hoehn KL, Turner N, Swarbrick MM, Wilks D, Preston E, Phua Y (2010). Acute or chronic upregulation of mitochondrial fatty acid oxidation has no net effect on whole-body energy expenditure or adiposity. Cell Metab.

[75] Cohen J, Collins R, Darkes J, Gwartney D (2007). A league of their own: demographics, motivations and patterns of 1,955 male adult non-medical anabolic steroid users in the United States. J Int Soc Sports Nutr.

[76] Monaghan LF (2002). Vocabularies of motive for illicit steroid use among bodybuilders. Social Sci Med.

[77] Christiansen AV, Bojsen-Møller J (2012). “Will steroids kill me if I use them once?” A qualitative analysis of inquiries submitted to the Danish anti-doping authorities. PEH.

[78] Boardley ID, Grix J (2014). Doping in bodybuilders: A qualitative investigation of facilitative psychosocial processes. Qual Res Sport Exerc Health.

[79] Boardley ID, Grix J, Dewar AJ (2014). Moral disengagement and associated processes in performance-enhancing drug use: a national qualitative investigation. J Sport Sci.

[80] Boardley ID, Grix J, Harkin J (2015). Doping in team and individual sports: a qualitative investigation of moral disengagement and associated processes. Qual Res Sport Exerc Health.

[81] Todd T (1987). Anabolic steroids: the gremlins of sport. J Sport Hist.

[82] Bandura A (1999). Moral disengagement in the perpetration of inhumanities. Pers Soc Psychol Rev.

[83] Pappa E, Kennedy E (2013). ‘It was my thought… he made it a reality’: Normalization and responsibility in athletes’ accounts of performance-enhancing drug use. Int Rev Sociol Sport.

[84] Bilard J, Ninot G, Hauw D (2011). Motives for illicit use of doping substances among athletes calling a national antidoping phone-help service: an exploratory study. Subst Use Misuse.

[85] Ohl F, Fincoeur B, Lentillon-Kaestner V, Defrance J, Brissonneau C. The socialization of young cyclists and the culture of doping. Int Rev Sociol Sport. 2013; doi: 1012690213495534.

[86] Overbye M, Knudsen ML, Pfister G (2013). To dope or not to dope: Elite athletes’ perceptions of doping deterrents and incentives. PEH.

[87] Walsh C (2011). Drugs, the Internet and change. J Psychoactive Drugs.

[88] Soussan C, Kjellgreen A (2014). Harm reduction and knowledge exchange – a qualitative analysis of drug-related Internet discussion forums. Harm Reduct J.

[89] Brennan R, Van Hout MC, Wells J (2013). Heuristics of human enhancement risk: a little chemical help?. Int J Health Promot Educ.

[90] Perry RJ, Zhang D, Zhang XM, Boyer JL, Shulman GI (2015). Controlled-release mitochondrial protonophore reverses diabetes and protonophore reverses diabetes and steatohepatitis in rats. Science.

[91] Advisory Council on the Misuse of Drugs. Prevention of drug and alcohol dependence. 2015. https://www.gov.uk/government/uploads/system/uploads/attachment_data/file/406926/ACMD_RC_Prevention_briefing_250215.pdf.

