# Supplementary material for: Russian roulette with unlicensed fat-burner drug 2,4-dinitrophenol (DNP): evidence from a multidisciplinary study of the internet, bodybuilding supplements and DNP users
Source: Subst Abuse Treat Prev Policy. 2015 Oct 14;10:39. doi: 10.1186/s13011-015-0034-1 (PMC4607104; doi:10.1186/s13011-015-0034-1)
Supplement: Additional file 1: — Method for the semi-quantitative screening for 2,4 dinitrophenol (DNP). (PDF 190 kb) [file 13011_2015_34_MOESM1_ESM.pdf]

Additional file 1: Method for the semi-quantitative screening for 2,4 dinitrophenol (DNP)

Materials

DNP (D198501-5 g), methanol (LC-MS grade), water (LC-MS grade), hexane (anhydrous, 95%), hydrochloric acid (ACS reagent, 37 %), formic acid and ammonium formate were also obtained from Sigma-Aldrich (Dorset, UK).

Instrumentation and LC-MS/MS analysis

The LC–MS/MS system comprised of a 1260 infinity LC system (Agilent Technologies UK) coupled to a 6430 triple quadrupole mass spectrometer (Agilent Technologies UK). The LC system consisted of a 1290 infinity thermo-stated autosampler, degasser, binary pump and column heater. An electrospray ionisation (ESI) source was used for samples analysis. The chromatographic separation was achieved using an Ascentis Express F5 (2.1 x 150 mm, 2.7 µm) column. The column was heated to 45°C for good reproducibility. The analytical column was connected in tandem with a 0.2 µm inline filter to prevent it from blocking. Mobile phase consisted of Solvent A [0.02 M formic acid in acetonitrile] and solvent B [10 mM ammonium formate/0.02 M formic acid in water]. Tried and tested LC-MS grade solvents were used for analysis.

The flow rate through the column was set at 0.4 mL/min. To obtain sharp peaks the following gradient flow was used. The mobile phase linear gradient was run with 90% solvent A for 0.5 minutes and 10% solvent B, decreasing to 50% solvent A in 1 minutes and the gradient was held at 50% solvent A from 1 to 2 minutes and then returned to 90% of solvent A until 3 minutes, and stabilised for further 2 minutes at 90% of solvent A before next injection. The injection volume was 10 µL. The bypass configurations were set up for the mixer and damper when using this kit with Agilent HPLC 1260 binary pump. This is to convert the pump to low delay volume mode and better chromatography. Negative electrospray ionization polarity was run for the analysis of DNP. Sheath gas temperature and flow was 350°C, 12 L/min, nozzle voltage was 450 V, drying gas temperature and flow 300°C, 8 L/min, nebulizer gas pressure was 25 psi and fragmentor voltage was set to 150 V, Delta retention time was 1 for all ions in dynamic MRM mode.

The Agilent MassHunter software was used for data generation. The total run time was 8 min together with 2 min post time. The MRM transitions, fragmentor voltages and collision energies of each analyte are given in Table 1. The product ions used as quantifier and qualifier ions in MS/MS analysis are highlighted in blue. Figure 1 shows an example for the LC-MS/MS chromatogram showing the DNP ions in a positive supplement sample.

Table 1. DNP precursor, product ions, collision energies, fragmentor voltages and abundances

**Negative ESI**			
**Precursor**	**Product**	**Fragmentor**	**Collision Energy**
**183.1**	**109.1**	**123**	**25**
	46.1	123	50
	**123**	**123**	**17**
	**137.1**	**123**	**17**

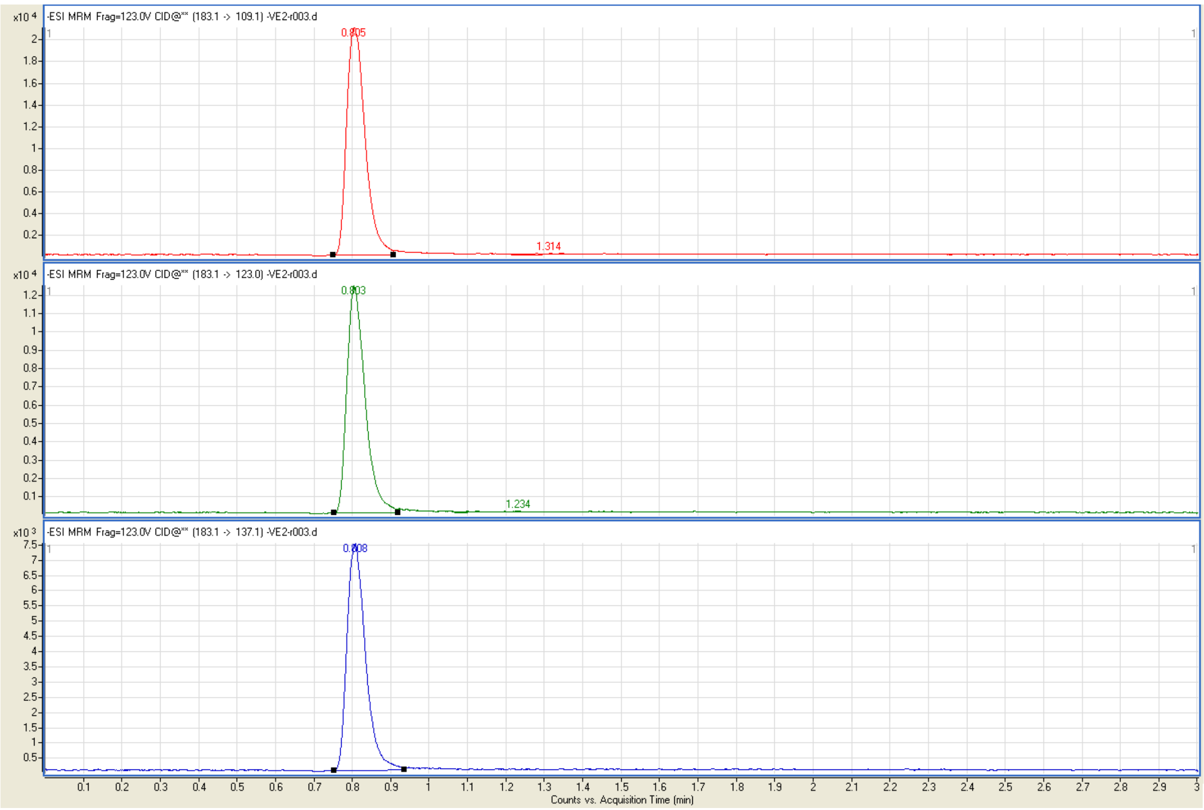

Figure 1. LC-MS/MS chromatogram showing DNP ions in a supplement sample.

Stock solution preparation. The stock solutions of DNP were prepared separately in methanol at a concentration of 1.00 mg/mL. Working solutions were prepared by serial dilution of the stock solution with 0.2 M HCl to obtain a set of standard concentrations. All working

standard solutions were stored at -20 °C and used within one month after preparation, although no significant degradation was observed in one month of storage.

Sample preparation and extraction. For extraction of DNP, the tablets, capsules and wax type supplements were left at room temperature, then individually cut into smaller parts and ground to a fine powder with a mini ball mill. Approximately 0.5 g of each tablet and capsule was weighed and mixed in 4 mL of 0.2 M HCl in a screw cap tube and vortexed well. The mixture was sonicated for extraction in an ultrasonic bath for 1 hour at 40°C. The homogenate was then cooled to room temperature and the volume was adjusted to the mark with 0.2 M HCl. This solution was centrifuged at 3500 × g for 10 min at 4 ºC. The supernatant was collected in a 10 mL glass tube with screw cap and 2 mL of hexane was added to this. The mixture was roller mixed for 10 min followed by centrifugation at 3500 × g (sample batch 1: high street) and at 2250 × g (sample batch 2: Internet) for 10 min. The lower aqueous layer was collected which was further purified using a 0.2 µm Millex® Syringe Filter Units (Non-sterile, 4/25mm) and 10 µL was injected onto an LC-MS/MS system. For wax type supplement, 0.5 g of sample was weighed in a 10 mL glass tube with screw cap and vortexed mixed with 2 mL of hexane. To this tube 4 mLs of 0.2 M HCl were added and roller mixed for 10 min at high speed. The lower aqueous layer was collected and filtered with a 0.2 µm Millex® Syringe Filter Units (Non-Sterile, 4/25 mm) and 10 µL was injected onto LC-MS/MS system.
