# Supplementary material for: Russian roulette with unlicensed fat-burner drug 2,4-dinitrophenol (DNP): evidence from a multidisciplinary study of the internet, bodybuilding supplements and DNP users
Source: Subst Abuse Treat Prev Policy. 2015 Oct 14;10:39. doi: 10.1186/s13011-015-0034-1 (PMC4607104; doi:10.1186/s13011-015-0034-1)
Supplement: Additional file 2: — List of retail web-based sites and discussion boards/forums. (PDF 389 kb) [file 13011_2015_34_MOESM2_ESM.pdf]

Additional file 2: List of retail web-based sites and discussion boards/forums

	URL	Type	Description	Geolocation of the IP address[a]
1	http://www.tradekey.com	Broker	Sodium crystal 200mg DNP capsules in a tamperproof container. Capsules are 00 size, yellow/white for crystal, green/white for powder, shipping capacity: 1000 capsule. Large discounts for all larger orders; price on request. Ships from UK.	Lansing, Michigan, USA
2	http://www.ecplaza.net/dnp-everything.html	Business to business	Offers several Chinese DNP suppliers	Seoul, Republic of Korea
3	http://www.anasci.org	Forum	Discussion threads on various aspects of DNP use and experiences	Orlando, Florida, USA
4	http://dinitrophenol.net	Forum	DNP user experience exchange with the explicitly stated aim to minimize harm	Montreal, Canada
5	http://forum.bodybuilding.com	Forum	Discussion threads on various aspects of DNP use and experiences	Boise, Idaho, USA
6	http://www.muscletalk.co.uk	Forum	Discussion threads on various aspects of DNP use and experiences	(IP address is between the Isle of Man and mainland UK)
7	http://www.professionalmuscle.com	Forum	Discussion threads on various aspects of DNP use and experiences	San Francisco, California, USA
8	http://thinksteroids.com	Forum	Discussion threads on various aspects of DNP use and experiences	New York, New York, USA
9	http://www.aboard.in	Forum	Discussion threads on various aspects of DNP use and experiences	Singapore, Malaysia
10	http://www.uglybodybuilding.com	Forum	Discussion threads on various aspects of DNP use and experiences	Amsterdam, The Netherlands
11	http://tnation.t-nation.com	Forum	Discussion threads on various aspects of DNP use and experiences	San Francisco, California, USA
12	http://evolutionary.org	Forum	Discussion threads on various aspects	San Francisco,

			of DNP use and experiences	California, USA
13	http://reddit.com/r/bodybuilding/	Forum	Discussion threads on various aspects of DNP use and experiences	San Francisco, California, USA
14	http://www.bodybuildindex.com	Forum	Discussion threads on various aspects of DNP use and experiences	Amsterdam, The Netherlands
15	http://www.uk-muscle.co.uk	Forum	Discussion threads on various aspects of DNP use and experiences	Innsbruck, Austria
16	http://www.bodybuildingdungeon.com	Forum	Discussion threads on various aspects of DNP use and experiences	San Jose, California, USA
17	http://www.mindandmuscle.net	Forum	Discussion threads on various aspects of DNP use and experiences	Sofia, Bulgaria
18	http://community.hypemuscle.com	Forum	Discussion threads on various aspects of DNP use and experiences	San Francisco, California, USA
19	http://www.canadabodybuilding.com	Forum	Discussion threads on various aspects of DNP use and experiences	Provo, Utah, USA
20	http://www.ugm.org.uk	Forum	Discussion threads on various aspects of DNP use and experiences	Leeds, UK
21	http://www.elitefitness.com	Forum	Discussion threads on various aspects of DNP use and experiences	Burnaby, Canada
22	http://www.bodybuildingforums.com.au	Forum	Discussion threads on various aspects of DNP use and experiences	Chicago, Illinois, USA
23	http://anabolicminds.com	Forum	Discussion threads on various aspects of DNP use and experiences	Dallas, Texas, USA
24	http://forums.isteroids.com	Forum	Discussion threads on various aspects of DNP use and experiences	Montreal, Canada
25	http://www.getbig.com	Forum	Discussion threads on various aspects of DNP use and experiences	Dallas, Texas, USA
26	https://www.tmuscle.co.uk	Forum	Discussion threads on various aspects of DNP use and experiences	Manchester, UK
27	http://www.worldclassbodybuilding.com	Forum	Discussion threads on various aspects of DNP use and experiences	Provo, Utah, USA
28	http://www.superiormuscle.com	Forum	Discussion threads on various aspects of DNP use and experiences	Dallas, Texas, USA

29	http://www.thevipboard.com	Forum	Discussion threads on various aspects of DNP use and experiences	Provo, Utah, USA
30	http://www.theironden.com	Forum	Discussion threads on various aspects of DNP use and experiences	Amsterdam, The Netherlands
31	http://juicedmuscle.com	Forum	Discussion threads on various aspects of DNP use and experiences	Bochum, Germany
32	http://projectbodybuilding.com	Forum	Discussion threads on various aspects of DNP use and experiences	Culver City, California, USA
33	http://forums.rxmuscle.com	Forum	Discussion threads on various aspects of DNP use and experiences	Beverly Hills, California, USA
34	http://www.bodybuilding-hardcore.com	Forum	Guidelines and information on DNP use	Scottsdale, Arizona, USA
35	http://www.rebodybuilding.com	Forum	Discussion threads on various aspects of DNP use and experiences	Kaunas, Lithuania
36	http://www.bodybuildingweb.net	Forum	Discussion threads on various aspects of DNP use and experiences	Houston, Texas, USA
37	http://www.pharmacyreviewer.com	Forum	Discussion threads on various aspects of DNP use and experiences	Wilmington, New Jersey, USA
38	http://forums.musculardevelopment.com	Forum	Discussion threads on various aspects of DNP use and experiences	Pine Brook, New Jersey, USA
39	https://testosteronekings.wordpress.com	Forum	Education and information blog	San Francisco, California, USA
40	http://brotherhoodofpain.com	Forum	Discussion threads on various aspects of DNP use and experiences	Plaza, Panama
41	http://www.wannabebig.com	Forum	Discussion threads on various aspects of DNP use and experiences	Lon Angeles, California, USA
42	http://www.ausbb.com	Forum	Discussion threads on various aspects of DNP use and experiences	Brea, California, USA
43	http://united-muscle.com	Forum	Discussion threads on various aspects of DNP use and experiences	Amsterdam, The Netherlands
44	http://www.bodybuildindex.com	Forum	Discussion threads on various aspects of DNP use and experiences	Amsterdam, The Netherlands
45	http://www.angelfire.com	Forum/ Information	Detailed dosing schedule	Waltham, Massachusetts, USA

46	http://www.anabolicextreme.com	Forum/Online Information	Discussion threads on various aspects of DNP use and experiences	Colombus, Ohio, USA
47	http://www.steroidology.com	Forum/Online library	Information on and discussion boards relating to DNP	Garden City, New York, USA
48	http://www.dnpweightloss.com	Online blog	Discussion threads on various aspects of DNP use and experiences	Springfield, Missouri, USA
49	http://www.basskilleronline.com	Online library	Article on DNP	Provo, Utah, USA
50	http://imbodybuilding.com	Online library	Article on DNP	Moscow, Russian federation
51	http://anabolics24.com	Retail	100mg Biomax (Biomax Labs, Turkey), 100 capsules for $80.00; Unknown location; ships to CA, ZA, EU, Asia, US	Bucharest, Romania
52	http://www.steroid.com/DNP.php	Retail	Does not sell DNP but gives reference to UnderGround Lab for availability (one 200mg pill for $1.00)	San Francisco, California, USA
53	http://dinitroshop.net	Retail	1 200mg powder capsule for $2.80	Cedar Knolls, New Jersey, USA
54	http://www.steroidmall.eu	Retail	Self-proclaimed "online pharmacy that provides secure transactions between patients and medical providers"; Sells in bulk; 224 Euros for 100 x 200mg capsules; contact information is for London, UK	(close to) Brasov, Romania
55	http://crystaldnp.com	Retail	Capsules (Sodium Salt, 250 mg/capsule for $1.00 per capsule); ships from Canada to AU, CA, EU, Other, UK, US	Hague, The Netherlands
56	http://myanabol.com	Retail	Resale website; 100mg ($195) or 200mg ($234) per 100 capsules, undisclosed location (possibly Turkey); ships to CA, EU, US; On custom seizure situations, one time reshipment for free; 98% delivery success claimed	(close to) Nicosia, Cyprus
57	http://www.absolutesteroids.com	Retail	200mg/capsule; 99% crystalline DNP	unavailable

			for $170.00; AU, CA, EU, US Products are by BR-Europe/Pumping Iron (now combined as UmForte); source is probably Turkey	
58	http://1buysteroids.net	Retail	250 mg/capsule for $4.00, ships worldwide from undisclosed location.	unavailable
59	http://buyinjectables.com	Retail	100 x 200mg capsule; 99% crystalline DNP for 129 Euros; shopts from undisclosed location to CA, EU, US	unavailable
60	http://www.buy-anabolic-steroids.com/en/	Retail	30 x 100mg capsules for $48; ships worldwide from undisclosed location.	Zurich, Switzerland
61	http://levram.us	Retail	Crystal, 25m capsules ($125.00); AbaXenPharmaceuticals; no longer sells DNP	Siauliai, Lithuania
62	http://www.isteroids.com	Retail	Does not sell DNP but provides information	Montreal, Canada
63	http://www.vpxsports.com	Retail	Does not sell DNP but provides information	Austin, Texas, USA
64	http://www.xroids.com	Retail	Does not sell DNP but provides information	Huerth, Germany
65	http://www.legitanabolics.net	Retail	60 x 200mg capsules for 120 Euros, Manufacturer: Bodyadvance Performance; claim that it ships from the European Union	Penang, Malaysia
66	http://buyabsolutesteroid.com	Retail	200 mg x 100 for $170; ships worldwide, prides in 98% success rate in shipping (except Germany) and offers a 'custom seize guarantee' except to Australia	Istanbul, Turkey
67	http://www.buy-clenbuterol.eu	Retail	200 mg x 11 capsules (one cycle) for $70; ships from Europe to worldwide via registered mail	Plovdiv, Bulgaria
68	http://www.roidsmall.net	Retail	30 x 100 mg capsules for $60;	Huerth, Germany

			manufacturer: Gen-Shi Laboratories, Japan; ships worldwide with reship guarantee except for Australia, Canada, New Zeeland, Singapore, South Korea	
69	http://www.anabolicsteroid-powder.com	Retail	Powder DNP, minimum order 100g, price is negotiated, origin: China	Seattle, Washington, USA
70	http://allsteroidsworld.com	Retail	100 mg capsules	Dronten, The Netherlands
71	http://www.ianabolicsteroids.com	Retail	Does not sell DNP but provides information and reference to the UnderGround Lab	
72	http://sellsteroids.en.made-in-china.com	Retail	Wholesale; US$ 1.0/kg 99% USP DNP powder (origin: China), production capacity: 1000 kg/month	San Antonio, Texas, USA
73	http://www.steroid.bz	Retail	Does not sell DNP but supply reference to the UnderGround Lab	Montreal, Canada
74	http://www.massroids.net	Retail	Gen-Shi Lab, 30 x 100 mg caps (packaging is labeled as supplement with L-Glutamin)	Huerth, Germany
75	http://www.isp.org.cn	Retail	Wholesale powder DNP, sold as "DNP anabolic steroid hormones"	Beijing, China
76	http://www.steroidmall.eu	Retail	100 x 200mg capsules for 224 Euros	(close to) Brasov, Romania
77	http://www.anabolic-steroids.biz	Retail	30 x 100 mg/caps, white tablets, for $60; manufacturer: Gen-Shi Labs; ships worldwide for $28 (shipping for order over $600 is free); reship guarantee except to reship the products only once. Be aware that we are not reshipping to the following locations: Australia, Canada, New Zeeland, Singapore, South Korea	Amsterdam, The Netherlands
78	http://www.steroidbazaar.com	Retail	100 x 200 mg capsules for $170.00;	Istanbul, Turkey

			ships worldwide, prides in 98% success rate in shipping (except Germany) and offers a 'custom seize guarantee' except to Australia	
79	http://drmalkins.en.com51.com	Retail	Seller is identified as "drmalkins" with a mailing address in Ukraine	Central district, Hong Kong
80	http://pharmaeu.net	Retail	100 capsules x 100mg for €33.65	Rotterdam, The Netherlands
81	http://www.neomeds.com	Retail	30 x 100mg/capsules (Gen-SHi Labs) for $60.00;	Huerth, Germany
82	http://umforteshop.com	Retail	25 x 100mg capsules for $57; authorised reseller of UmForte products;	Southfield, Michigan, USA
83	http://buydnponline.com	Retail	25 x 100mg capsules for $57; authorised reseller of UmForte products;	Southfield, Michigan, USA
84	http://www.daddyroids.com	Retail	30 x 100mg (Gen-Shi Lab) for $60.00;	Huerth, Germany
85	http://cheaperpharma.com	Retail	50 x 200mg capsules (origin brand: Signature Pharmaceuticals, Greece) for $60.00	Rotterdam, The Netherlands
86	http://www.musclechemistry.com	Retail/Forum	Does not sell DNP but runs discussion	(close to) Engelberg, Switzerland
87	http://ec91141043.sell.curiousexpeditions.org	Retail	Place of origin: China<br>Powder, price quoted, minimum order 100g<br>Discrete shipping	Beijing, China
88	http://anabolicsteroidpowder.sell.nccecojustice.org/	Retail	Place of origin: China<br>Chrystal, price negotiable, minimum order 100g; Discrete shipping	Zurich, Switzerland
89	http://www.vjirdv.com/	Retail	HongKong Bodybuilding Biopharmaceutical Co.,Ltd<br>Powder (wholesale), price quoted, Place of origin China; Minimum order:	Los Angeles, USA
90				
	http://www.shuzixizang.com/			San Francisco, USA

			10g; supply ability :5000kg/month; Shipment worldwide with reship policy; 100% pass to UK, Norway, Poland, Spain, USA, Canada, Brazil; 98% pass to Germany, Russia, Australia and New Zealand.	
91	http://rawsteroidpowder.sell.everychina.com/	Retail	Shenzhen Shijingu Technology Co., Ltd. Crystal; Place of origin China; Minimum order: 10g; supply ability :500000kg/month	Beijing, China
92	http://m.steroid-supplement.com/	Retail	Powder; Place of origin Hong Kong; Minimum order: 100g; supply ability :1000kg/month	Dallas, USA

[a] IPligence™ online free IP geolocator (http://www.ipligence.com/geolocation) was used to search for the geographical location of the website IPs
